# The chromosome 11q13.3 amplification associated lymph node metastasis is driven by miR-548k through modulating tumor microenvironment

**DOI:** 10.1186/s12943-018-0871-4

**Published:** 2018-08-21

**Authors:** Weimin Zhang, Ruoxi Hong, Lin Li, Yan Wang, Peina Du, Yunwei Ou, Zitong Zhao, Xuefeng Liu, Wenchang Xiao, Dezuo Dong, Qingnan Wu, Jie Chen, Yongmei Song, Qimin Zhan

**Affiliations:** 1 Key laboratory of Carcinogenesis and Translational Research (Ministry of Education/Beijing), Laboratory of Molecular Oncology, Peking University Cancer Hospital & Institute, Beijing, 100142 China; 2 State Key Laboratory of Oncology in South China, Collaborative Innovation Center of Cancer Medicine, Sun Yat-Sen University Cancer Center, Guangzhou, 510060 China; 3 BGI Genomics, BGI-Shenzhen, Shenzhen, 518083 Guangdong China; 4 Department of Neurosurgery, Tiantan Hospital, Capital Medical University, Beijing, 100050 China; 5 State Key Laboratory of Molecular Oncology, Cancer Institute and Cancer Hospital, Chinese Academy of Medical Sciences and Peking Union Medical College, Beijing, 100021 China; 6 Institute of Cancer Stem Cell, Cancer Center, Dalian Medical University, Dalian, 116044 China; 7 Shanghai Clinical Center for Endocrine and Metabolic Diseases, Shanghai Key Laboratory for Endocrine Tumours, Rui-Jin Hospital, Shanghai Jiao-Tong University School of Medicine, Shanghai, 200240 China

**Keywords:** Lymphatic metastasis, Lymphangiogenesis. Tumor microenvironment miR-548k, Esophageal squamous cell carcinoma

## Abstract

**Background:**

The prognosis for esophageal squamous cell carcinoma (ESCC) patients with lymph node metastasis (LNM) is still dismal. Elucidation of the LNM associated genomic alteration and underlying molecular mechanisms may provide clinical therapeutic strategies for ESCC treatment.

**Methods:**

Joint analysis of ESCC sequencing data were conducted to comprehensively survey SCNAs and identify driver genes which significantly associated with LNM. The roles of miR-548k in lymphangiogensis and lymphatic metastasis were validated both in vitro and in vivo. ESCC tissue and blood samples were analyzed for association between miR-548k expression and patient clinicopathological features and prognosis and diagnosis.

**Results:**

In the pooled cohort of 314 ESCC patients, we found 76 significant focused regions including 43 amplifications and 33 deletions. Clinical implication analysis revealed a panel of genes associated with LNM with the most frequently amplified gene being MIR548K harbored in the 11q13.3 amplicon. Overexpression of miR-548k remarkably promotes lymphangiogenesis and lymphatic metastasis in vitro and in vivo. Furthermore, we demonstrated that miR-548k modulating the tumor microenvironment by promoting VEGFC secretion and stimulating lymphangiogenesis through ADAMTS1/VEGFC/VEGFR3 pathways, while promoting metastasis by regulating KLF10/EGFR axis. Importantly, we found that serum miR-548k and VEGFC of early stage ESCC patients were significantly higher than that in healthy donators, suggesting a promising application of miR-548k and VEGFC as biomarkers in early diagnosis of ESCC.

**Conclusions:**

Our study comprehensively characterized SCNAs in ESCC and highlighted the crucial role of miR-548k in promoting lymphatic metastasis, which might be employed as a new diagnostic and prognostic marker for ESCC.

**Electronic supplementary material:**

The online version of this article (10.1186/s12943-018-0871-4) contains supplementary material, which is available to authorized users.

## Background

Esophageal squamous cell carcinoma (ESCC) is one of the most common aggressive and lethal malignancies in the world and especially in China, where ESCC is the fourth leading cause of cancer-related mortality [1]. Due to the limitation of clinical approaches for early diagnosis and treatment, the 5-year survival rate of ESCC is still dismal, ranging from 10 to 25% [2]. The high rates of local invasion and lymphatic metastasis are main reasons for poor clinical outcome of patients with ESCC. It is considered that regional lymph node metastasis (LNM) is the initial event of tumor cells dissemination in ESCC [3, 4]. Although LNM is a widely employed independent prognostic indicator, and often guides therapeutic decisions in ESCC, the underlying molecular mechanisms are yet far from fully understood.

Tumor cells have evolved two main sophisticated manners for spreading to regional lymph nodes, including eliciting lymphangiogenesis by secreting lymphangiogenic growth factors and invading pre-existing lymphatic vessels in the tumor periphery [3–5]. Lymphangiogenesis is a complex cellular process involving proliferation, sprouting, migration and tube formation [6]. The proliferation and migration of lymphatic endothelial cells (LECs) in tumor microenvironment were driven by the VEGFC/VEGFR3 axis. Tumor cells secreted VEGFC and activated VEGFR3 of LECs to provoke the growth of lymphatic vessels [7, 8]. Accumulating evidence has revealed that VEGF-C is overexpressed and positively correlated with lymphatic vessels density and LNM in a variety of malignancies, including breast cancer, colorectal cancer, lung cancer, gastric cancer and ESCC [9–13]. Importantly, disrupting the conversation of tumor cells and LECs by interferring RNA or neutralizing antibodies to VEGFC or VEGFR3, has been shown to reduce the rate of lymph node metastasis in vitro and in vivo [14, 15]. Excitingly, VGX-100, a VEGFC monoclonal antibody, has been evaluated efficacy in a phase I clinical trial for advanced or metastatic solid tumors (NCT01514123) [16]. These findings suggests that VEGFC/VEGFR3 axis plays a crucial role in lymphangiogenesis and lymphatic metastasis. Therefore, understanding the regulatory mechanisms of VEGFC in ESCC may provide clinically valuable predictive tools for effective anti–VEGFC treatments.

Copy number alterations (CNAs) can confer substantial phenotypic plasticity and have been described as the driving force of genetic diversification [17]. There is evidence supporting a greater role for CNAs rather than somatic mutations in initiation and progression of cell malignant transformation [17, 18]. Genome-wide profiling of CNAs in ESCC has been reported in previous studies, helping to understand the extent and distribution of CNAs in the ESCC genome. Integrating these identified CNA regions and functional knowledge of the affected genes with clinic-pathological parameters have revealed several diagnostic and prognostic significant recurrent CNAs and genes in ESCC. Intriguingly, in previous studies, we and other groups have reported that overexpression of ORAOV1, ANO1 and FADD in accordance with 11q13.3 amplicon were revealed to be positively correlated with LNM [1, 19–22]. It seems that 11q13.3 amplicon could serve as an indicator for the presence of LNM. However, despite the clinical association, the causal relationship and underlying molecular mechanisms of 11q13.3 amplicon involved in lymph node metastasis are still largely unknown. Interestingly, in this region, we identified a microRNA named miR-548k and demonstrated its genetic variation and biological functions for the first time [1]. Recently, miR-548k was reported to regulated ESCC cell proliferation through targeting long noncoding RNA-LET (lncRNA-LET) [23] Jinpin Li et al. demonstrated that miR-548k played an important role in myasthenia gravis pathogenesis by downregulated the expression of CXCL13 [24]. Given that one miRNA could target different mRNAs to exert diverse functions, it is reasonable to speculated that miR-548k may act as a molecular driver of 11q13.3 amplicon associated with LNM.

For an uncharacterized gene, the extent or significance of CNAs is often used to evaluate its relevance to cancer [25]. However, a major challenge is to distinguish the alterations that play causative roles (drivers) from the random alterations (passengers) that accumulate during esophageal carcinogenesis [26]. Thus, a compelling need exists to yield higher resolution data and extensively identify precision breakpoints for each CNA in ESCC, elucidating its molecular basis to guide the development of early diagnosis and precise targeted therapies. In this study, we conducted a joint analysis of four Chinese ESCC cohorts and integrated the TCGA data to systematically study the CNAs and the related genes in ESCC, with particular focus on the association of lymph node metastasis and the underlying mechanisms. We found a panel of CNAs associated genes implicated with LNM and *MIR548K*, which is harbored in 11q13.3 amplicon, being the most significant gens with the highest frequency of amplification. We demonstrated that overexpression of miR-548k remarkably promotes cell proliferation, lymphangiogensis and lymphatic metastasis in ESCC in vitro and in vivo, through regulating ADAMTS1/VEGFC/VEGFR3 pathway and KLF10/EGFR axis. Importantly, the serum level of miR-548k in early stage ESCC patients were significantly higher than that in healthy donators and higher in patients with LNM than those without LNM in the same cohort. Therefore, our study reveal a comprehensive LNM associated CNAs genes and propose a potential causal link between the chromosomal 11q13.3 amplification and lymph node metastasis for the first time.

## Methods

### Copy number alterations analysis

This study pooled the individual genomic data of subjects from four independent whole genome sequencing (WGS) and whole exome sequencing (WES) of esophageal squamous cell carcinoma [1, 2, 27, 28]. Copy number alterations analysis was performed as described [29]. Briefly, copy number alterations (CNAs) were detected with SegSeq for 31 WGS, and GATK4 Alpha for 283 WES. GISTIC2.0 [30] was performed to identify significantly amplified or deleted genomic regions. A total of 84 genomic regions were obtained, and 76 focused regions exhibited significant amplification or deletion (*q* < 0.1, Additional file 1: Table S1). Genes harbored in focused regions that frequency of copy number gain or loss > = 20% were selected for further analysis.

Fisher’ exact test was used to calculate the association between copy number alteration and lymph node metastasis, *p* < 0.05 was considered statistically significant. The Spearman’s Rank Correlation Coefficient was used to calculate the association of RNA expression versus DNA copy number in TCGA ESCC cohort. The survival rate was calculated by the Kaplan-Meier method, and the difference was compared by the Log-rank method. Cox proportional hazards model was used for the analysis of hazards, as implemented in the R package ‘survival’ (http://cran.r-project.org/web/packages/survival/). We removed the patients whose survival information were unavailable. By univariate analyses, the significance of the clinical variables was *p* < 0.1 level, and the significance of clinical multivariates was *p* < 0.05.

### Transwell migration assays

Transwell migration assays was performed as described in our previous study [31]. Briefly, migration of cells was assayed in Transwell cell culture chambers with 6.5 mm diameter polycarbonate membrane filters containing 8 μm pore size (Neuro Probe, Gaithersburg, MD, United States). In total, 1 × 105 HDLEC cells in 100 ul of serum-free medium were added to the upper chamber of the device, and the lower chamber was filled with 600 μL conditioned media (added 20% FBS) of KYSE30-Lenti-miR-548k cells or control cells. After 4 to 10 h of incubation at 37 °C, the non-migration cells were removed from the upper surface of the membrane with a cotton swab. The filters were then fixed in methanol for 10 min, stained with crystal violet solution for 1 h, and counted. Five random microscopic fields (× 100) were counted per well and the mean was determined.

### Conditioned media preparation

Conditioned media preparation assay was performed as described previously [31]. Briefly, cells were grown on 100 mm plates in about 70~ 80% confluence and then transformed to serum free media for 12 h. Cells were collected and counted, the cell ratio of different group were recorded. Conditioned media were collected after centrifugation at 2000 rpm at the temperature of 4 °C for 10 min. After being centrifuged at 12,000 rpm for 20 min, the supernatants were stored at 4 °C. For secretory proteins Western Blot assay, the supernatants were ultrafiltered with an Amicon Ultra-10 K device (Millipore) at 5000 rpm for 20 min. Concentrated media were normalized of the cell number of different groups and analyzed by Western blot analysis.

### Tube formation assay

Tube formation assay was performed as discribed in the paper of Libing Song and their colleagues [32]. Briefly, the human dermal lymphatic endothelial cells (HDLECs) tube formation assay was performed by first pipetting 50 μL 30% Matrigel (BD Biosciences, Bedford, Massachusetts, USA) into each well of a 96-well plate, which was then polymerised for 4 h at 37 °C. HDLECs (1 × 10^4^) in 100 μL of conditioned medium (cultural supernatant of miR-548k overexpression cells or control cells, respectively) were added to each well and incubated at 37 °C, 5% CO2 for 12 h. Images were taken using a bright-field microscope at × 100 magnification. The capillary tubes were quantified by measuring the total numbers of completed tubule structures.

### Luciferase reporter assay

Luciferase activity assay were performed to test whether miR-548k binding to the 3’-UTRs of KLF10 and ADAMTS1 mRNAs. Briefly, cells were collected 24 h after transfection and analyzed by using the Dual-Luciferase reporter assay system (Promega, Madison, WI). Luciferase activity was measured by Synergy H1 microplate fluoroscence reader (BioTek, U.S.A). The pRL-TK plasmid with constitutive expression of Renilla luciferase was co-transfected with different firefly luciferase-based reporter as internal control.

### Xenograft tumor formation and metastasis assay in nude mice model

Xenograft tumor formation and metastasis assay in nude mice model were performed as described previously [33]. For xenograft tumor formation study, 2 × 106 KYSE30-Lenti-miR-548k cells and control cells were injected subcutaneously into the right and left dorsal flank, respectively, of BALB/c nude mice (fourteen mice per group). Over a one month period, tumor formation in nude mice was observed by measuring the tumor volume calculated by the formula: π × 4/3 × larger diameter × smaller diameter square. Tumors were then excised and embedded in paraffin for haematoxylin and eoson staining (H&E) and immunohistochemistry (IHC) analysis. All animal experiments were approved by the Committee on the Use of Live Animals in Teaching and Research, Cancer Institute and Hospital, Chinese Academic of Medical Sciences & Peking Union Medical College.

For esophaguse in situ xenograft tumor formation study, 6 to 7 weeks old BALB/c nude mice (six mice per group, body weight ± 2 g) were narcotized and applied to abdominal surgery, then 2 × 105 KYSE30-Lenti-miR-548k cells and control cells were transplanted KYSE30-Lenti-miR-548k cells and their control cells into subserosa of esophageal abdominal portion by micro-injector (Hamilton 700, Switzerland). Two mice in the control group were died during anesthesia. Two months after injection, all mice were sacrificed and esophaguses were dissected for bioluminescence imaging and HE staining.

For metastasis assay, 5 × 105 cells (KYSE30-Lenti-miR-548k cells and control cells) were injected intravenously through the tail vein into 5 to 6 weeks old BALB/c nude mice (night mice per group). After 2 months, lungs were excised and embedded in paraffin for further study.

### Popliteal lymph node metastasis assay

Popliteal lymph node metastasis assay was performed as discribed in the paper of Libing Song and their colleagues [32]. Briefly, BALB/c-nu mice (female, 5–6 weeks old, 18–20 g) were randomly divided into two groups (*n* = 9/group). The miR-548k and GFP stable expression KYSE30 cells (5 × 10^5^) were inoculated into the foot-pads of the mice. The mice were sacrificed after half and a month, and the primary tumors and popliteal lymph nodes were collected and paraffin embedded. Serial 4.0 μm sections were taken and analyzed by IHC with anti-LYVE-1 (Abcam) and anti-GFP (Santa Cruz) antibodies.

### Statistical analysis

Statistical analysis was carried out using IBM SPSS Statistics 20 or GraphPad Prism 5 for Windows. Two tailed Student’s t-test was used to analyze the results expressed as Mean ± S.E.M. The two tailed Pearson χ^2^ test was used to analyze the association of miR-548k expression and clinic-pathological parameters. The survival curves were plotted by using Kaplan-Meier analysis and compared by log-rank test. Survival data were evaluated by univariate and multivariate Cox regression analysis. ROC (receiver operator characteristic) curve was used to determine the diagnostic accuracy. Differences were considered significant when the *p* value was less than 0.05.

## Results

### LNM associated somatic SCNAs across 314 ESCCs

**Fig. 1:**
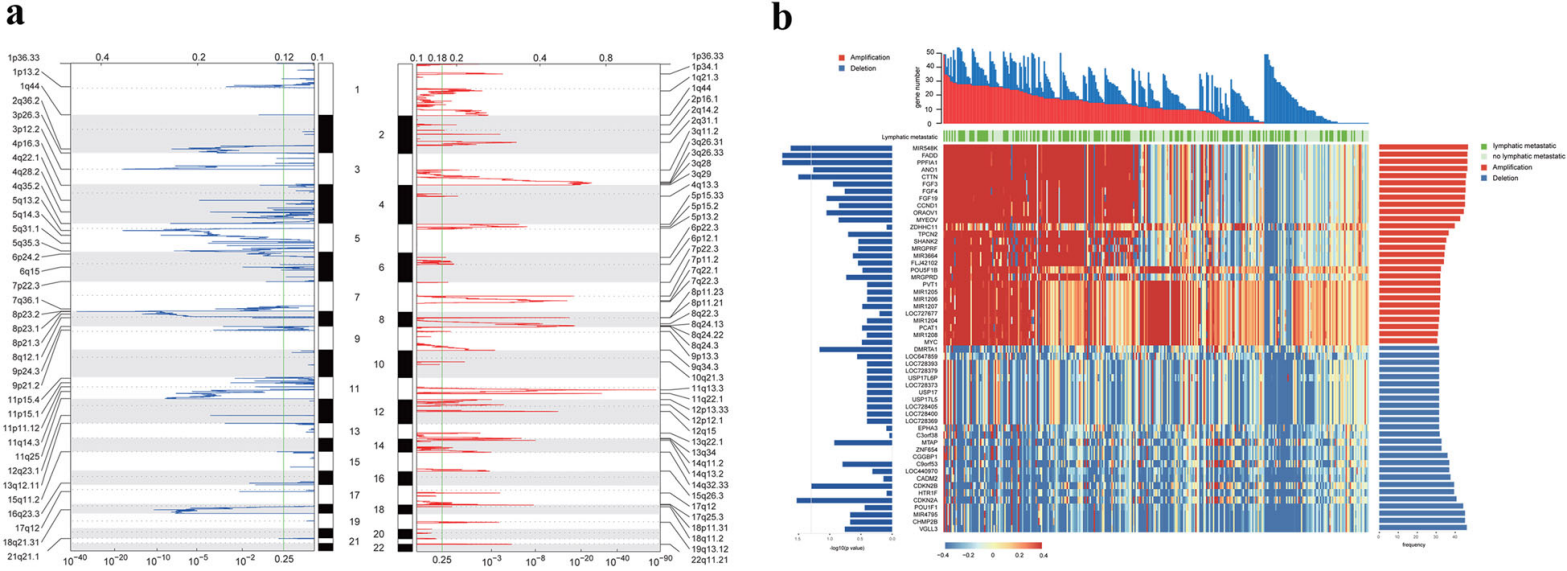
Somatic copy number alterations analysis in pooled ESCC cohort. **a**, Significance of SCNAs recurrences across chromosomes. **b**, The frequency and lymph node metastasis implication of SCNAs harbored genes. Genes of alteration frequency more than 30% were shown here. The upper panel shown the number of alteration genes in each sample. The middle panel shown the lymph node metastasis (LNM) status of each sample. The bottom heatmap shown the copy number of the indicated genes in each sample. The left panel shown the log10 (*p* value) of LNM association of each gene. The right panel shown the alteration frequency of each gene

To comprehensively profile the SCNAs in ESCC, we pooled our previous sequencing data with additional data from two other groups [1, 2, 27, 28]. SegSeq were used to detect SCNAs in 31 WGS data and GATK4 Alpha for 283 WES. GISTIC2.0 [30] was employed to identify significantly amplified or deleted genomic regions. A total of 84 genomic regions were obtained, and 76 focused regions exhibited significant amplification or deletion (q < 0.1, Fig. 1a and Additional file 1: Table S1). The most significant amplification and deletion regions were 11q13.3 and 8p23.1, respectively (Fig. 1a and Additional file 1: Table S1). Peaks involving important cancer genes such as *CCND1, EGFR, ERBB2, FGFR1, AKT1, MYC, KRAS* and *CDKN2A/2B*, which were confirmed in our data (Additional file 1: Table S1). As SCNAs have been known to play an important role in gene expression regulation, joint analysis of SCNAs and expression data of the resident genes should provide more information for detecting driver genes in ESCC tumorigenesis. Genes whose mRNA expression changes were in accordance with the corresponding CNAs might most likely to exert the functions of SCNAs. Although the samples in our pooled cohort have not been examined the expression of SCNAs harbored genes, we inferred the TCGA ESCC cohort as compromise for screening a panel of genes that SCNAs were in accord with the mRNA expression level. This approach identified 257 genes in our cohort among 404 alterations genes with frequency more than 20%, suggesting these genes might exert critical biological functions in the developing of ESCC (Additional file 1: Table S4). Pathway-based analysis approaches provided additional insights into the underpinnings molecular mechanisms of ESCC. Thus, the 257 genes were subjected to pathway enrichment analysis using GeneAnalytics [34], and the results revealed that Hippo pathway, TNF signaling, Wnt-mediated β-catenin signaling, TGF-beta receptor signaling pathway, TP53 network, and regulation of activated PAK-2p34 by proteasome mediated degradation pathway were most affected dysregulating pathways in ESCC (Additional file 1: Table S5).

**Fig. 2:**
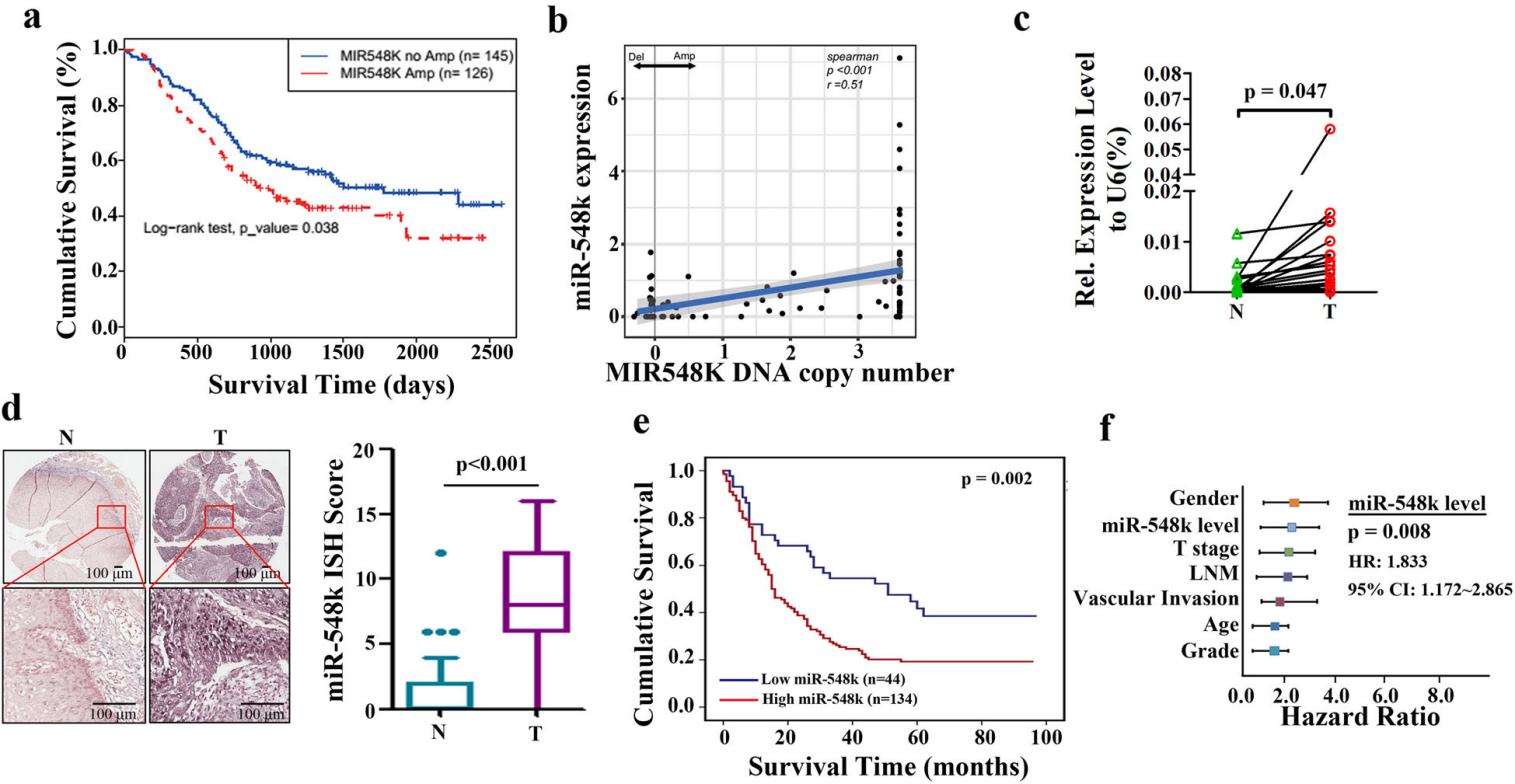
Clinical implication of miR-548k. **a**, Kaplan-Meier survival analysis of pooled ESCC cohort stratified by miR-548k amplification (*n* = 314; *p* = 0.038, log-rank test). **b**, The association of miR-548k RNA expression versus DNA copy number in TCGA ESCC cohort (*p* < 0.001, *r* = 0.51, Spearman’s Rank Correlation Coefficient analysis). **c**, Relative expression levels of miR-548k in 23 pairs of human ESCC tissues and matched adjacent normal tissues. **d**, Left, representative miRNA ISH photos of miR-548k expression in ESCC tissues and matched adjacent normal tissues. Scale bar: 100 μm. Right, MiRNA in situ hybridization (ISH) score in ESCC tissues and matched adjacent normal tissues (*n* = 185). **e**, Kaplan-Meier survival analysis of all ESCC patients stratified by miR-548k expression level (*n* = 178; *p* = 0.002, log-rank test). **f**, Multivariate analysis of the hazard ratios (HR) showed that the upregulation of miR-548k may be an independent prognostic factor for the overall survival rate (by the Cox multivariate proportional hazard regression model). The HR is presented as the means (95% confidence interval, 95% CI)

Regional lymph node metastasis is well accepted prognostic and diagnostic factor in patients with ESCC, which was considered as an early step for cancer dissemination and progression [35, 36]. However, the mechanisms that control lymph node metastasis are unclear. To dissect the potential mechanisms from the genomic perspective, we analyzed the association of SCNAs harbored genes with lymph node metastasis. In our result, there were 28 genes exhibited significantly related to lymph node metastasis. Among the top significantly associated genes, *RTP4, RTP2, MASP1, BCL6, FLJ42393, LOC100131635, SLC2A2, LPP* were identified to correlated with LNM for the first time (Additional file 1: Tables S2 and S3). The most frequent alteration genes associated with LNM were *MIR548K, FADD, PPFIA1, CTTN* and *CDKN2A,* which were almost existed in 11q13.3 (Additional file 1: Table S2). It seems that 11q13.3 amplicon could serve as an indicator for the presence of LNM. Despite the clinical association, the underlying molecular mechanisms of 11q13.3 amplicon involved in lymphatic metastasis are still largely unknown. Among the LNM associated genes, *MIR548K* was the most frequent amplified genes that account for 46.82% of patients (Fig. 1b, Additional file 1: Table S2). Additionally, *MIR548K* amplification was significantly correlative with poor survival outcome of patients with ESCC (Fig. 2a). Given the fact that each miRNA can regulate hundreds of mRNAs to mediate diverse biological functions, dysregulation of miRNAs are intimately related to tumorigenesis. *MIR548K* might exert the driver role of the 11q13.3 amplicon in lymphatic metastasis in ESCC.

### Frequently overexpression of miR-584k in human ESCC tissues

To investigate whether copy number alterations of *MIR548K* could result in mRNA overexpression, we first analyzed the TCGA ESCC cohort and confirmed that CNVs of *MIR548K* were significantly positive correlated with miR-548k mRNA level (*r* = 0.51, *p* < 0.001, Fig. 2b). We then examined the expression level of miR-548k in 23 pairs of ESCC tissues and their matched adjacent normal tissues using real time PCR. As shown in Fig. 2c, miR-548k was significantly up-regulated in ESCC tissues compared to their matched adjacent normal tissues. Consistently, miRNA In situ Hybridization Histochemistry (miRNA ISH) assay also confirmed that miR-548k was overexpression in ESCC tumor tissues (Fig. 2d). These findings indicate that miR-548k is up-regulated in human ESCC.

### Up-regulated miR-548k expression predicts aggressive clinicopathological characteristics and poor prognosis in ESCC patients

We further explored the association between miR-548k expression level and different clinicopathological features of patients with ESCC. In 185 pairs paraffin-embedded, archived ESCC tissues and matched adjacent normal tissues cohort, statistical analyses revealed that miR-548k expression level was significantly correlated with American Joint Committee on Cancer (AJCC) stage (*p* = 0.005), patient overall survival (*p* = 0.005) and lymph node metastasis (LNM, *p* = 0.040, Additional file 1: Table S7).

Notably, miR-548k overexpression strongly associated with poor survival of ESCC patients (*p* = 0.002, Kaplan–Meier survival analysis and log-rank test, Fig. 2e), and the 5-year survival rate in the miR-548k high expression group (13.43%) was substantially lower than that of the miR-548k low expression group (34.09%). The median survival in the miR-548k low expression group was 51 months (95%CI 17.925 to 84.075), while 15 months (95%CI 11.455 to 18.545) in the miR-548k high expression group. Multivariate Cox regression survival analysis adjusting for age, vascular invasion, T stage, LNM, pathological grade, gender and miR-548k level consistently reported strong correlation between miR-548k overexpression and shorter survival (*p* = 0.008, HR = 1.833, 95% CI 1.172 to 2.865, Fig. 2f, Additional file 1: Table S16), indicating that miR-548k expression was an independent prognostic factor for outcome in ESCC. In fact, the stratification by miR-548k level displayed even higher prognostic significance than the widely employed LNM (*p* = 0.005, HR = 1.681, 95% CI 1.172 to 2.412, Fig. 2f, Additional file 1: Table S16).

### Oncogenic roles of miR-548k in ESCC

Previously, we have demonstrated that transient overexpression miR-548k mimics promoted, whereas miR-548k inhibitors suppressed ESCC cells growth, colony formation, migration and invasion in vitro [1]. Here, we used lentivirus to generate two stable overexpressed miR-548k cell lines KYSE30-Lenti-miR-548k and KYSE510-Lenti-miR-548k (Additional file 2: Figure S1a) to further confirm these findings. Consistently, we found that stable overexpression of miR-548k substantially increased the ability of ESCC cell growth, colony formation and cellular motility and invasiveness and promoted G_2_/M phase cell cycle progression (Additional file 2: Figure S1b-g).

**Fig. 3:**
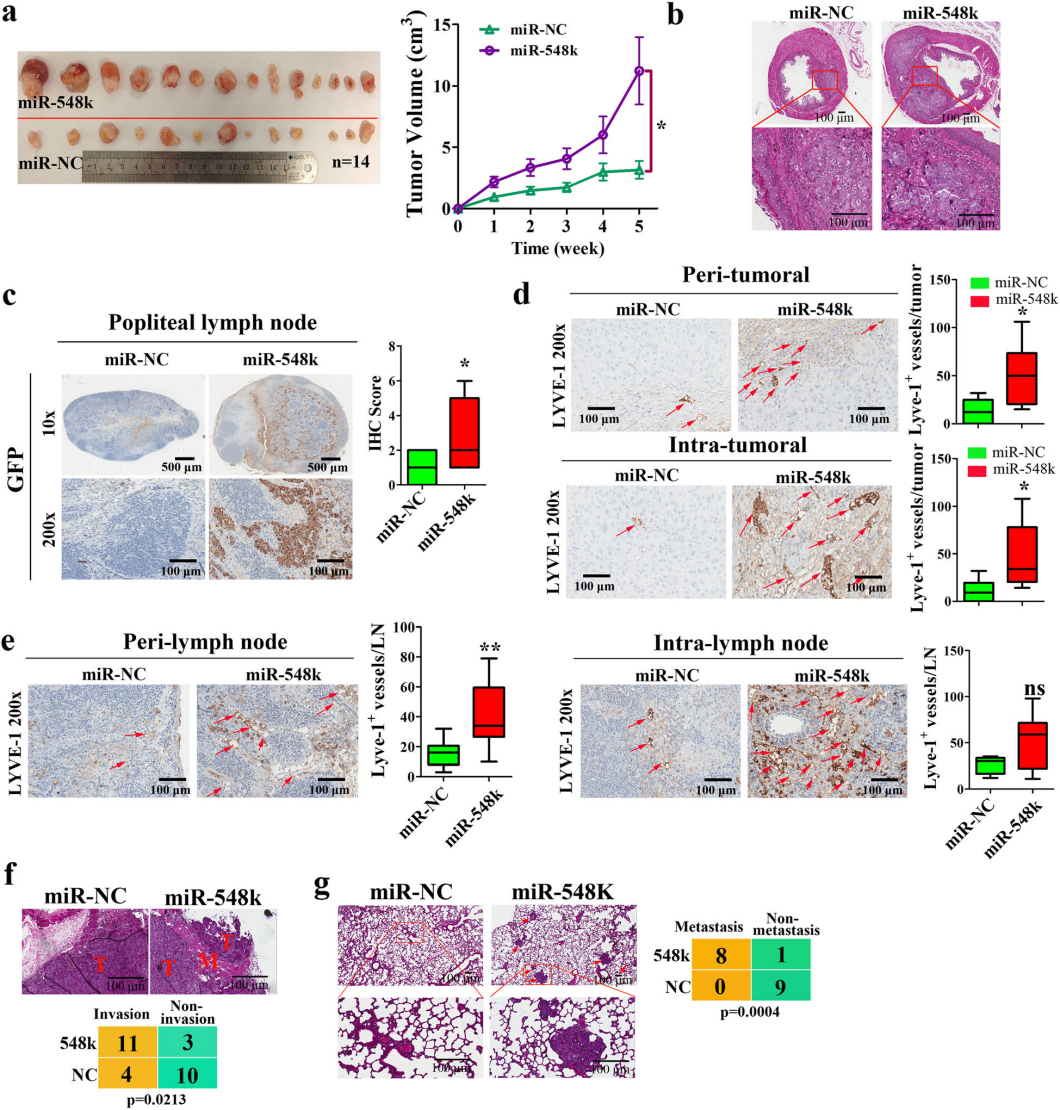
In vivo mouse model studies reveal miR-548k promoted xenograft tumor formation, lymphangiogenesis, lymphatic metastasis and distant metastases*.* **a**, Stable overexpression of miR-548k in KYSE30 cells enhanced subcutaneous xenograft tumors formation in BALB/c nude mice (*n* = 14). Left, representative picture. Right, growth curves of xenograft tumors. **b**, Stable overexpression of miR-548k in KYSE30 cells promoted subserosa tumor growth in esophageal abdominal portion (*n* = 6 in miR-548k overexpression group and *n* = 4 in control group). Representative HE photos of esophageal subserosa tumors. Scale bar: 500 μm or 100 μm. **c**, Representative images and quantitative analysis of the popliteal lymph nodes immunostained with anti-GFP antibody (*p < 0.05, t-test). Scale bar: 500 μm or 100 μm. **d**, Representative images of peri-tumoral and intra-tumoral sections immunostained with anti-LYVE-l antibody (left), and quantification (right), indicates the microlymphatic vessel density (*p < 0.05, t-test). Scale bar: 100 μm. **e**, Representative images of peri-lymph node and intra-lymph node sections immunostained with anti-LYVE-l antibody (left), and quantification (right), indicates the microlymphatic vessel density (**p < 0.01, ns, no significance, t-test). Scale bar: 100 μm. **f**, MiR-548k promoted local invasion in subcutaneous xenograft tumors formation model (*p* = 0.0213, Fisher’s Exact-test). Representative pictures (up) and quantitative data (bottom), T, tumor; M, muscle. Scale bar: 500 μm. **g**, Stable overexpression of miR-548k in KYSE30 cells promoted the lung metastasis (*p* = 0.0004, Fisher’s Exact-test). Representative pictures (up) and quantitative data (bottom). Scale bar: 500 μm or 100 μm

To determine the biological roles of miR-548k in human ESCC progression in vivo, we conducted different nude mice models. Firstly, KYSE30-Lenti-miR-548k cells or KYSE510-Lenti-miR-548k paired with their counterparts were injected subcutaneously to the right flank and left flank of BALB/c nude mice to allow xenograft tumor formation, respectively. The volume of tumors was measured at the indicated time point and mice were sacrificed five weeks after implantation. As a result, the tumor xenografts in miR-548k stable overexpression group grew rapidly and were significantly larger than the control group (Fig. 3a, Additional file 2: Figure S2a). The proliferation markers PCNA and Ki-67 were consistently higher expressed in the miR-548k overexpression xenografts than that in the control group (Additional file 2: Figure S2b, c). Additionally, we also performed abdominal surgery and transplanted KYSE30-Lenti-miR-548k cells and their control cells into subserosa of esophageal abdominal portion for subserosa tumor growth analysis in the BALB/c nude mice by microinjection. Then mice were sacrificed and esophaguses were dissected for bioluminescence imaging and HE staining two months after injection. As shown in Additional file 2: Figure S2d, the GFP fluorescence intensity in KYSE30-Lenti-miR-548k cells group was significantly higher than the control group. Meanwhile, the HE assay also shown that miR-548k overexpression cells were grown more rapidly in esophagus compared to the control cells (Fig. 3b). These findings clearly show that miR-548k promotes ESCC cell proliferation, cell cycle progression, and enhances the development of ESCC malignancy.

### MiR-548k promotes ESCC lymphangiogensis and lymph node metastasis

Since 11q13.3 amplicon was involved in lymphatic metastasis and miR-548k overexpression was significantly correlated with lymphatic metastasis, we further investigated the role of miR-548k in the promotion of lymphangiogenesis in ESCC. Transwell migration assay and tube formation assay revealed that conditioned media of miR-548k overexpression remarkably provoked while inhibition significantly repressed the ability of ESCC cells to induce migration and tube formation in human dermal lymphatic endothelial Cells (HDLECs, Additional file 2: Figure S3a-d), indicating that miR-548k promotes ESCC lymphangiogenesis in vitro.

To confirm the above observations, we used a popliteal lymph node metastasis model to explore the effect of miR-548k on lymphangiogensis and lymphatic metastasis in vivo. KYSE30-Lenti-miR-548k cells and their counterparts, which stably expressed GFP, were inoculated into the foot-pads of nude mice (*n* = 9/group). Four weeks after first inoculation, the foot-pad xenograft tumors and popliteal lymph nodes were enucleated and analyzed. We found that the lymph nodes in tumors formed from miR-548k stable overexpression cells had larger volumes than tumors formed from vector-control cells (Additional file 2: Figure S4a, b). As expected, all the foot-pad xenograft tumors expressed GFP (Additional file 2: Figure S4c) and the lymph nodes in tumors formed from miR-548k high expression cells displayed more GFP-positive tumor cells than tumors formed from control cells (Fig. 3c). Interestingly, the tumor formed by KYSE30-Lenti-miR-548k cells shown increased levels of microlymphatic vessel density (MLD) compared with the control tumors, both peri-tumoral and intra-tumoral tissues, as indicated by the LYVE-1-positive microvessels (Fig. 3d). Meanwhile, we also found that levels of MLD were increased in peripheral popliteal lymph nodes from the flanks of tumors formed from miR-548k stable overexpression cells compared to the control flanks (Fig. 3e, left). Though it had no significant statistical differences, the levels of MLD were seem to be increased in intra-lymph nodes from the flanks of tumors formed from miR-548k stable overexpression cells compared to the control flanks (Fig. 3e, right). Taken together, these findings suggest that miR-548k promotes lymphangiogenesis and lymph node metastasis in ESCC in vivo.

### MiR-548k provokes metastasis of ESCC cells in vivo

LN metastasis is a complex multistep process [6]. In addition to intratumoral and peritumoral lymphangiogenesis, enhanced cell invasion and migration are essential for metastasis [37, 38]. As described above, miR-548k overexpression promoted cell mobility and invasiveness (Additional file 2: Figure S1f, g). To elucidate the metastatic promotion potential of miR-548k in vivo, we evaluated the regional invasiveness of the xenograft tumors derived from KYSE30-Lenti-miR-548k cells and their control cells. As a result, the invasive capacity of the KYSE30-Lenti-miR-548k cells was significantly greater than the control cells (Fig. 3f), 78.6% (11/14) of the miR-548k overexpression xenograft tumors have infiltrated into the peripheral muscle tissue, while the control group was just 28.6% (4/14). Furthermore, tail vein tumor cells (KYSE30-Lenti-miR-548k cells or control cells) injection assay was conducted to test whether overexpression of miR-548k could promote the the metastatic ESCC cell colonization of the lung. After two months, mice were sacrificed and their lungs were subjected to further analysis. HE staining and statistics analysis showed that the metastatic rate of miR-548k overexpression cells was substantially higher than the control cells (88.9% vs 0.0%, *p* = 0.0004; Fig. 3g). Collectively, these findings indicated that miR-548k increases the mobility and invasiveness of ESCC cells in vivo.

### MiR-548k targets ADAMTS1 to facilitate ESCC lymphangiogensis

**Fig. 4:**
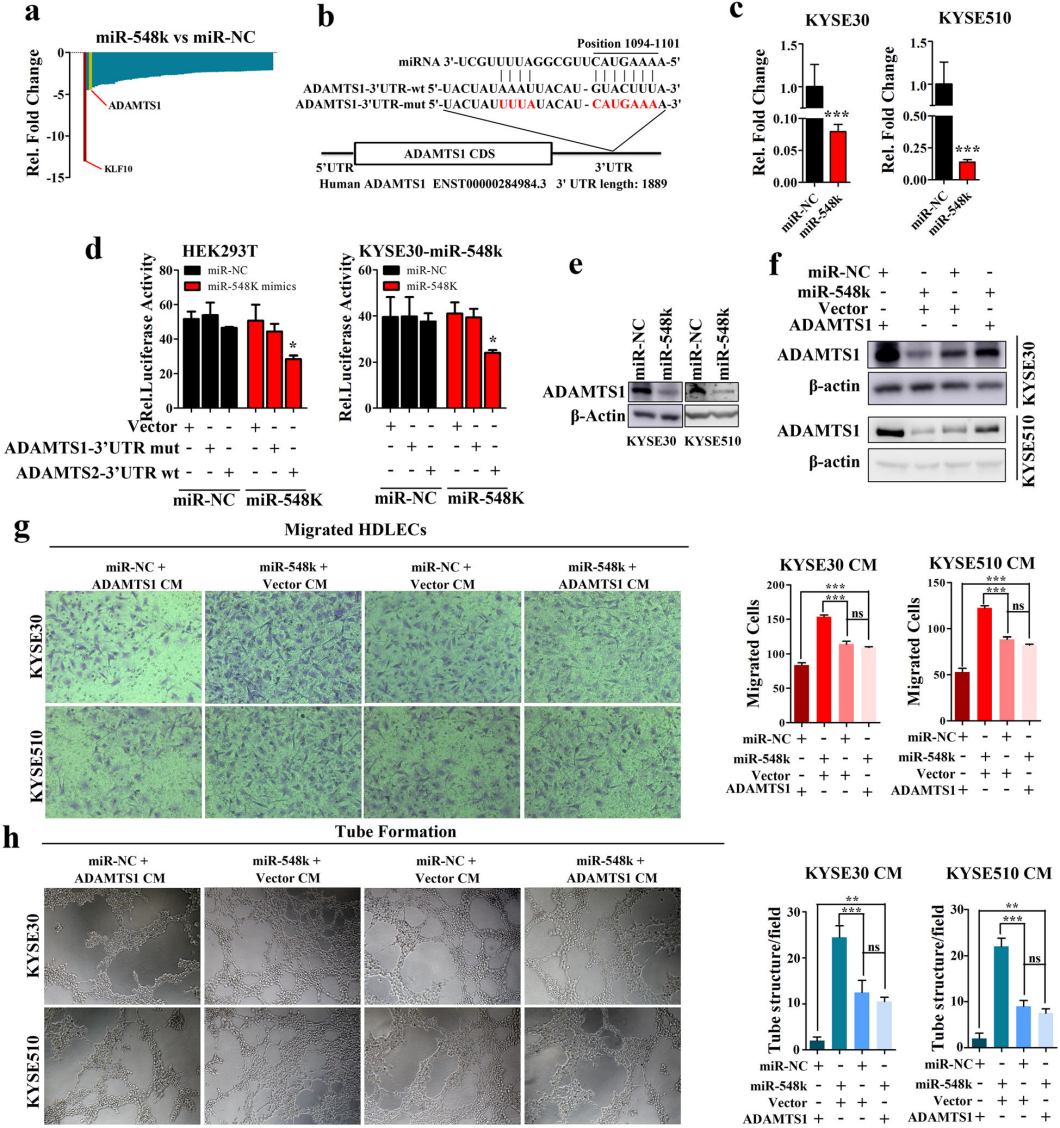
ADAMTS1 is a direct target of miR-548k in ESCC. **a**, Candidate targets of miR-548k. **b**, The predicted binding sequence of human hsa-miR-548k and its binding site in the 3′-untranslated region (3’-UTR) of ADAMTS1 were presented for alignment. ADAMTS1–3’-UTR-mut was the mutated sequences of 3’-UTR of ADAMTS1 without the binding sites of miR-548k. **c**, Quantitative real time PCR results show that ADAMTS1 could be downregulated KYSE30 cells (left) and KYSE510 cells (right) (*p* < 0.001, t-test). Data were expressed as mean ± S.E.M of three independent experiments. **d**, Luciferase assay was used to confirm the interaction of miR-548k with ADAMTS1. 3’-UTR of ADAMTS1 containing the target binding site (ADAMTS1–3’-UTR-wt) and the mutated sequences of 3’-UTR of ADAMTS1 (ADAMTS1–3’-UTR-mut) were cloned into downstream of a firefly luciferase gene. The plasmids were transfected into empty vector and miR-548k stably expressing cells (KYSE30-lenti-miR-548k). Renilla luciferase plasmid was co-transfected for normalisation. GV126-Control vector was co-transfected as positive control (*p < 0.05). **e**, Western blot analysis the expression level of ADAMTS1 in miR-548k overexpression cells and control cells. **f**, Ectopic expressed ADAMTS1 open reading frame plasmid without 3’-UTR (cannot be targeted by miR-548k) in miR-548k overexpression cells (KYSE30-Lenti-548 k and KYSE510-Lenti-548 k) and control cells. **g** and **h**, Representative images (left) and quantitative data (right) of HDLECs cultured with conditioned medium derived from miR-548k overexpressing cells and control cells with or without ectopic expression of ADAMTS1. **g**, Transwell migration assays, left representative images; right, quantitative data. **h** Matrigel tube formation assay, left representative images; right, quantitative data. CM, conditioned medium. Error bars represent the mean ± S.E.M from three independent experiments, ns, no significance

To explore the molecular mechanisms underlying miR-548k-induced lymphatic metastasis in ESCC, we integrated bioinformatic analysis and mRNA microarray assay to investigate the potential targets of miR-548k. To this end, we first searched the online microRNA target prediction tool TargetScan [39] and performed mRNA microarray assay on miR-548k overexpression cells and the control cells, then obtained lymph node metastasis associated genes from the GeneCards database (http://www.genecards.org) with keyword ‘inhibit lymph node metastasis’, integrating these three gene sets and yielding a panel miR-548k target candidates (Fig. 4a, Additional file 2: Figure S5). According to gene ontology annotation, we selected ADAMTS1 (a disintegrin and metalloproteinase with thrombospondin motifs 1) for further study. Given that ADAMTS1 was implicated in lymphangiogensis [40], we hypothesized miR-548k regulating the lymphatic metastasis through targeting ADAMTS1. Our analysis revealed that the 3’-UTR of ADAMTS1 mRNA contains a complementary site for the seed region of miR-548k, which is conservative across different species (Fig. 4b and Additional file 2: Figure S6a). Quantitative real time PCR assays confirmed that ADAMTS1 was remarkably downregulated upon miR-548k overexpression in KYSE30 and KYSE510 (Fig. 4c). To verify whether or not ADAMTS1 is direct target of miR-548k, a fragment containing the miR-548k binding site of ADAMTS1 wild type 3’-UTR and the mutant (222 bp, Fig. 4b) cloned into a vector with the firefly luciferase reporter gene. As a result, luciferase activity was significantly reduced in ADAMTS1 3’-UTR-wt transfected cells compared to the ADAMTS1 3’-UTR-mut or empty vector controls in both HEK293T cell line and KYSE30-Lenti-miR-548k cell line (Fig. 4d), which ensured the specificity of binding between miR-548k and the 3’-UTR of ADAMTS1. Additionally, the protein level of ADAMTS1 was decreased as consequence of miR-548k ectopic overexpression (Fig. 4e). Taken together, these results suggest that ADMATS1 is a regulated target of miR-548k.

Next, we asked whether miR-548k regulating lymphangiogensis in ESCC was partially dependent on downregulating of ADAMTS1. ADAMTS1 open reading frame plasmid without 3’-UTR (cannot be targeted by miR-548k) was introduced in the miR-548k stable overexpression ESCC cells, and the conditioned media were collected. Interestingly, ectopic expression of ADAMTS1 could reduce the abilities of miR-548k on migration and tube formation in HDLECs (Fig. 4f-h). Consistently, the condition media of ADAMTS1 knockdown cells induced more rapidly and more tube formation in HDLECs than the control media, while media from silencing ADAMTS1 and simultaneously inhibiting miR-548k cells attenuated these abilities compared to miR-548k inhibition along in KYSE150 cells (Additional file 2: Figure S7 and Additional file 2: Figure S8a, b). Taken together, these findings further confirm the biology functions of miR-548k on lymphangiogensis.

### MiR-548k modulates tumor microenvironment via ADAMTS1/VEGFC/VEGFR3 pathway

**Fig. 5:**
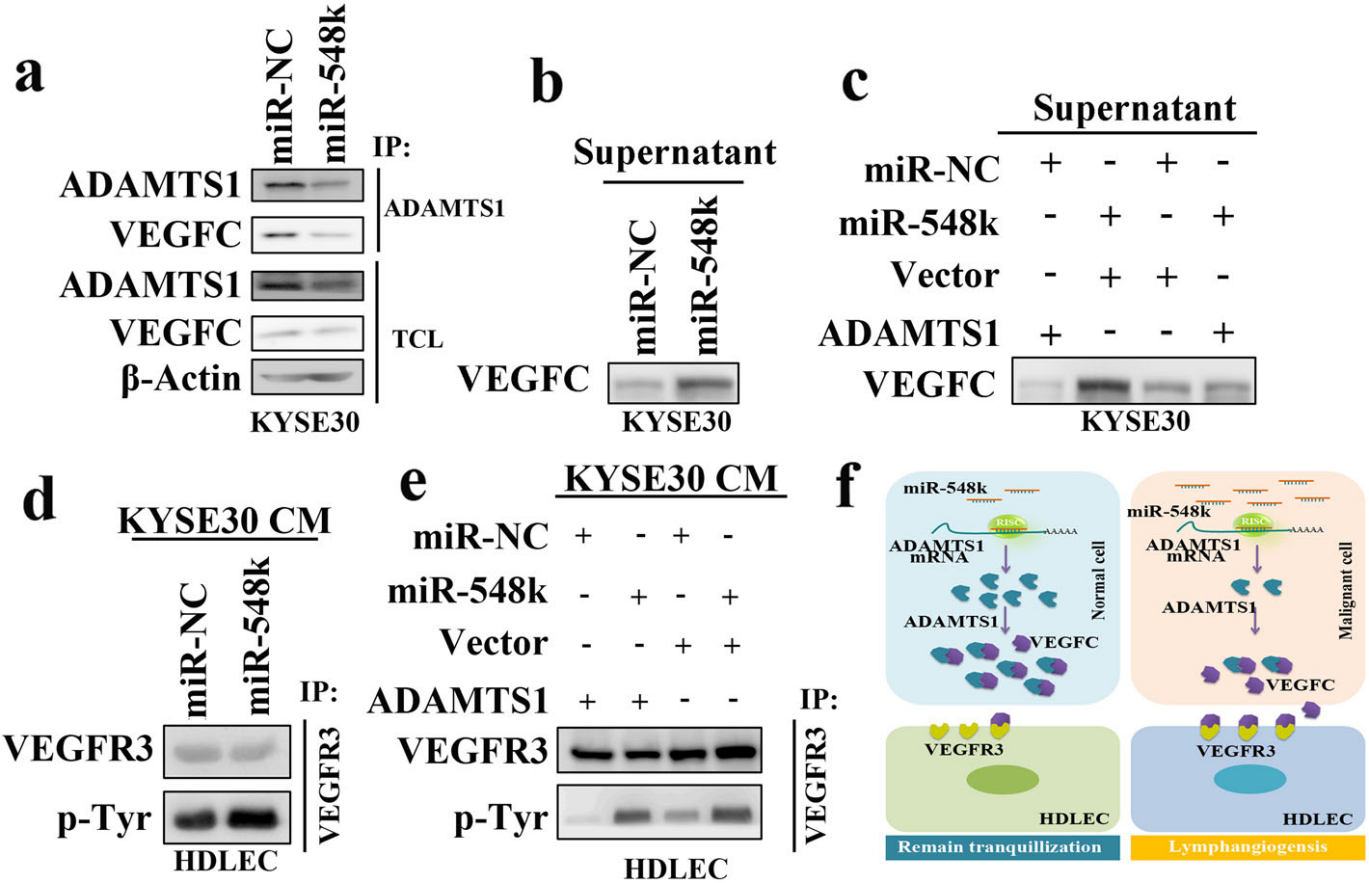
The effect of miR-548k on VEGFR3 phosphorylation. **a**, Immunoprecipitation and Western blot assays were used to examine the interaction of ADAMTS1 and VEGFC in miR-548k overexpression cells (KYSE30-Lenti-miR-548k) and control cells. Total cell lysates (TCL) of miR-548k overexpression cells and control cells were immunoprecipitated with the antibody against the indicated proteins. Immunocomplexes were then immunoblotted using antibodies against the indicated proteins. TCL were also immunoblotted using antibodies against the indicated proteins. **b**, The secretory VEGFC was examined in supernatants of miR-548k overexpression cells and control cells. **c**, The secretory VEGFC was examined in supernatants of ADAMTS1 and or miR-548k overexpression cells and control cells. **d**, The phosphorylation level of VEGFR3 in HDLEC cells. HDLEC cells were cultured in conditioned media of miR-548k overexpression cells and control cells. Cells were immunoprecipitated with the antibody against VEGFR3. Immunocomplexes were then immunoblotted using antibodies against the indicated proteins. **e**, The phosphorylation level of VEGFR3 in HDLEC cells. HDLEC cells were cultured in conditioned media of ADAMTS1 and/or miR-548k overexpression cells and control cells. Cells were immunoprecipitated with the antibody against VEGFR3. Immunocomplexes were then immunoblotted using antibodies against the indicated proteins. **f**, Illustrative model showing the proposed mechanism by which miR-548k promotes cell lymphangiogenesis and lymphatic metastasis in ESCC via suppressing ADAMTS1 expression

Previous study has shown that ADAMTS1 could physically interact with VEGFC in breast cancer cells [40]. Consistently, our result also confirmed the interaction between ADAMTS1 and VEGFC in ESCC cells (Fig. 5a). Maybe due to the suppression effect of miR-548k on ADAMTS1, the ADMATS1/VEGFC protein complex was less in miR-548k overexpression cells than the control cells (Fig. 5a). Interestingly, we detected more secretory VEGFC in miR-548k overexpression cells than the control cells (Fig. 5b). To test weather miR-548k promoted VEGFC secretion was via downregulation the expression of ADAMTS1, we ectopic expressed ADAMTS1 in miR-548k overexpression cells and control cells. All cells were cultured with the same amount of media and in the same culture conditions. Cell culture supernatants were collected and concentrated, than normalized of the cell number of different groups and analyzed by western blot. As shown in Fig. 5c, secretory VEGFC was decreased upon ADAMTS1 rescue. Taken together, these findings suggest that miR-548k can suppress ADAMTS1 expression and release more amounts of free VEGFC from ADAMTS1 sequestration.

Given that VEGFC/VEGFR3 signaling is essential for lymphangiogensis, we sought to determine whether the effect of miR-548k on lymphangiogensis was via VEGFR3 activation. Firstly, we cultured HDLEC cells with conditioned media of miR-548k overexpression cells and control cells. In line with expectations, miR-548k promoted tyrosine phosphorylation (p-Tyr) of VEGFR3 in HDLECs (Fig. 5d), while ADAMTS1 rescue attenuated the p-Tyr level of VEGFR3 (Fig. 5e). Collectively, our results reveal that overexpression miR-548k may promote lymphangiogensis and lymphatic metastasis of ESCC through modulating the conversation between tumor cells and lymphatic endothelial cells in tumor microenvironment via ADAMTS1/VEGFC/VEGFR3 cascade (Fig. 5f).

### KLF10-EGFR pathway is potentially involved in miR-548k-regulated ESCC cell metastasis

After shaping a favorable pre-metastatic microenvironment, the critical step for LN metastasis is to activate the mobility and invasiveness associated signaling pathways. Interestingly, our analysis revealed that KLF10, one of the EGFR transcriptional repressors, was a miR-548k target candidate (Fig. 4a). Given the fact that EGFR was a key regulator of tumor metastasis, we sought to elucidate whether miR-548k promoting lymphatic metastasis was through mediating the EGFR signaling pathways.

**Fig. 6:**
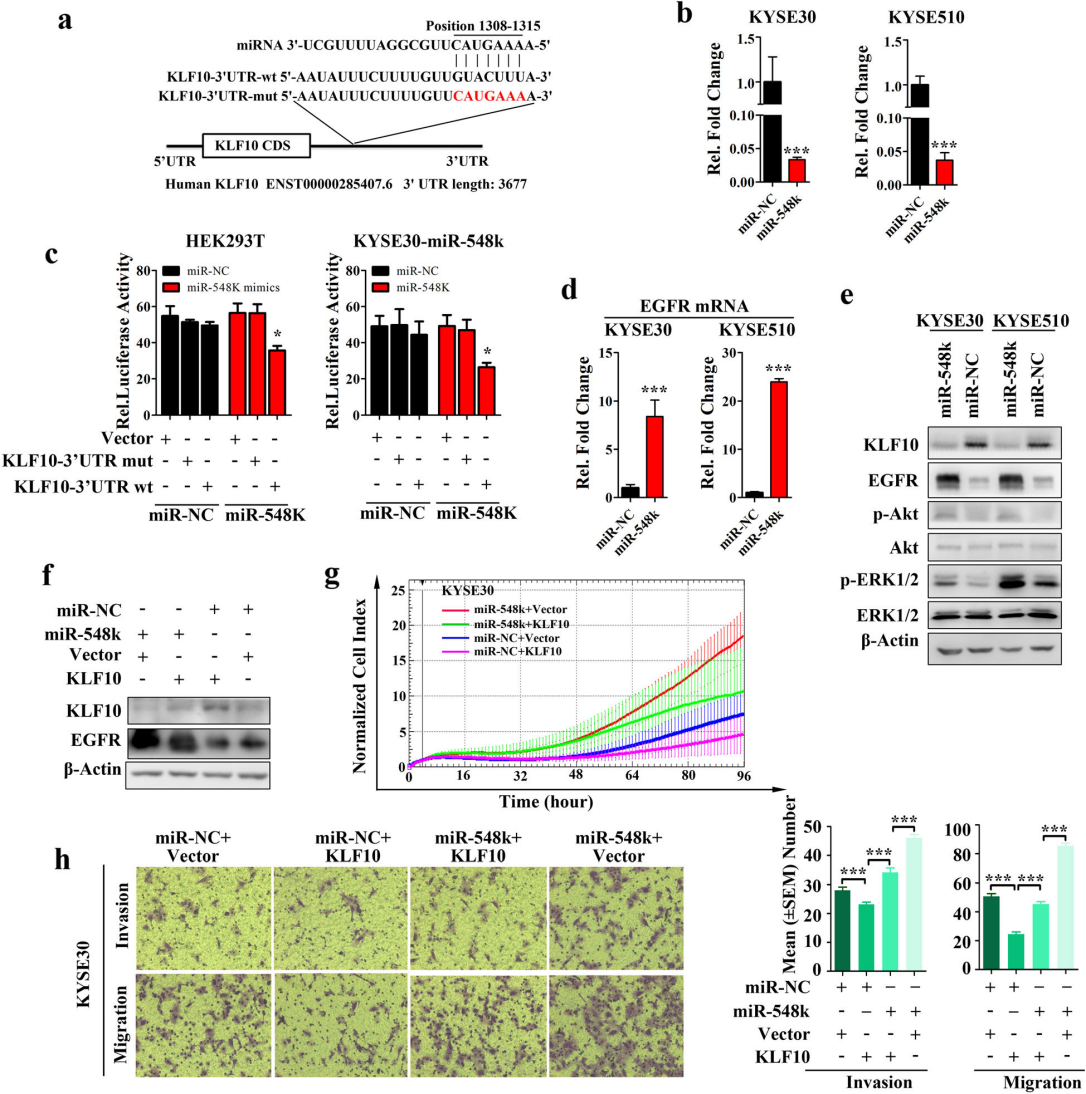
MiR-548k modulates EGFR pathways by directly targeting KLF10 in ESCC. **a**, The predicted binding sequence of human hsa-miR-548k and its binding site in the 3′-untranslated region (3’-UTR) of KLF10 were presented for alignment. KLF10–3’-UTR-mut was the mutated sequences of 3’-UTR of KLF10 without the binding sites of miR-548k. **b**, Quantitative real time PCR results show that KLF10 could be downregulated by miR-548k in KYSE30 cells and KYSE510 cells (*p* < 0.001, t-test). Data were expressed as mean ± S.E.M of three independent experiments. **c**, Luciferase assay was used to confirm the interaction of miR-548k with KLF10. 3’-UTR of KLF10 containing the target binding site (KLF10–3’-UTR-wt) and the mutated sequences of 3’-UTR of KLF10 (KLF10–3’-UTR-mut) were cloned into downstream of a firefly luciferase gene. The plasmids were transfected into empty vector and miR-548k stably expressing cells (KYSE30-lenti-miR-548k). Renilla luciferase plasmid was co-transfected for normalisation. GV126-Control vector was co-transfected as positive control (*p < 0.05). **d**, Real time PCR examined the mRNA level of EGFR in miR-548k overexpression cells (KYSE30-Lenti-548 k and KYSE510-Lenti-548 k) and control cells. **e**, Western blot analysis the expression level of KLF10, EGFR and the phosphorylation protein level and total protein level of ERK1/2, Akt in miR-548k overexpression cells and control cells. **f**, Ectopic expressed KLF10 open reading frame plasmid without 3’-UTR (cannot be targeted by miR-548k) in miR-548k overexpression cells (KYSE30-Lenti-548 k and KYSE510-Lenti-548 k) and control cells. **g**, Growth curves of miR-548k overexpressing cells (KYSE30-Lenti-miR-548k) and control cells (KYSE30-Lenti-miR-NC) with or without ectopic expression KLF10 open reading frame. **h**, Transwell assays evaluated the migration and invasion capacities of miR-548k overexpressing cells (KYSE30-Lenti-miR-548k) and control cells (KYSE30-Lenti-miR-NC) with or without ectopic expression KLF10 open reading frame. Left, representative images; right, quantitative data. Error bars represent the mean ± S.E.M from three independent experiments, ns, no significance

KLF10 contains putative target sequence of miR-548k (Fig. 6a, Additional file 2: Figure S6b). To validate the in-silico prediction, a serial of assays were used to test whether the expression of KLF10 could be repressed by miR-548k. Real time PCR assay confirmed that KLF10 mRNA was downregulated in KYSE-30-Lenti-miR-548k cell line and KYSE-510-Lenti-miR-548k cell line compared to their counterparts (Fig. 6b). To further evaluate whether KLF10 is the direct downstream target of miR-548k, a fragment of the 3’UTR of KLF10 (222 bp) containing the wild type or mutation potential miR-548k binding site (KLF10 3’UTR-wt, or KLF10 3’UTR-mut, respectively) was cloned into a vector with the firefly luciferase reporter gene (Fig. 6a). Luciferase activity was significantly reduced in KLF10 3’UTR-wt transfected cells compared to the KLF10 3’UTR-mut or empty vector controls in both HEK293T cell line and KYSE-30-Lenti-miR-548k cell line (Fig. 6c), which ensured the specificity of binding between miR-548k and the 3’UTR of KLF10. Previous studies have demonstrated that KLF10 was a transcriptional repressor of EGFR. Thus, we would like to investigate the relationship between miR-548k and EGFR pathway. As expected, EGFR mRNA was upregulated accompany with miR-548k overexpression (Fig. 6d). Consistently, the protein level of KLF10 was decreased, whereas the protein of EGFR was increased upon ectopic overexpression of miR-548k (Fig. 6e). These results suggest that miR-548k may regulate the EGFR expression through targeting KLF10. Interestingly, the downstream pathways of EGFR, including Akt and ERK1/2, were activated subsequently with miR-548k overexpression, the phosphorylation level of these proteins were significantly upregulated (Fig. 6e). In tumor xenograft formed from miR-548k overexpression cells, EGFR and phosphorylation of Akt were consistently higher than that in control counterparts (Additional file 2: Figure S9).

To determine the relative contribution of KLF10-EGFR pathway to the miR-548k implicated malignances, we performed a serial KLF10 rescue assay with or without miR-548k overexpression. The data shown that ectopic expression of KLF10 coding sequence (CDS) region could compromise the phenotypes of miR-548k overexpression, which including reducing cell proliferation, migration and invasion (Fig. 6f-h). Consistently, knockdown KLF10 increased the cellular mobility in KYSE150 cells, while silencing KLF10 and simultaneously inhibiting miR-548k attenuated the ability of migration and invasion compared to miR-548k inhibition along in KYSE150 cells (Additional file 2: Figure S10a, b). Collectively, our findings indicate that miR-548k could enhance the capacity of mobility and invasiveness of ESCC cells by downregulating KLF10 and activating EGFR pathway subsequently.

### The clinical relevance and prognostic value of miR-548k and its target genes in ESCC

**Fig. 7:**
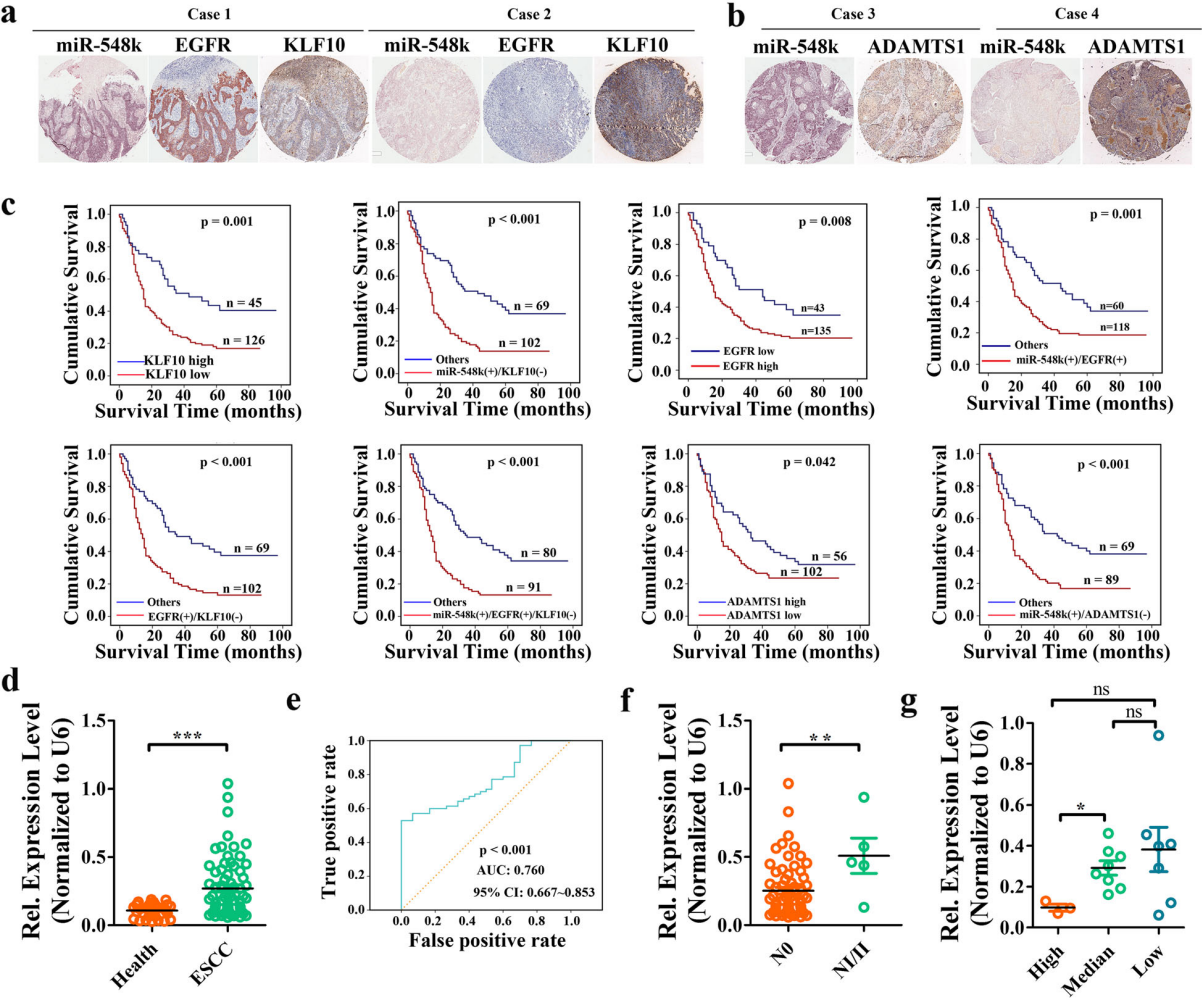
Up-regulated miR-548k expression levels predict aggressive clinicopathological characteristics and a poor prognosis in ESCC patients. **a**, Representative miRNA ISH photos of miR-548k expression and EGFR, KLF10 IHC images in the same ESCC samples. Scale bar: 100 μm. **b**, Representative miRNA ISH photos of miR-548k expression and ADAMTS1 IHC images in the same ESCC samples. Scale bar: 100 μm. **c**, Kaplan–Meier survival analysis (log-rank test) and multivariate Cox analysis of all ESCC patients stratified by different clasifiers as indicated. **d**, The abundance of miR-548k in serum of early ESCC patients and health people were detected by real time PCR. Relative expression level were normalized to U6. **e**, ROC curve evaluated the diagnostic accuracy of miR-548k abundance. ROC, receiver operator characteristic. AUC, area under the curve. **f**, MiR-548k abundance in the serum of ESCC patients with or without lymph node metastasis. **g**, MiR-548k abundance in the serum of ESCC patients with different grade of differentiation. ***p < 0.001. **p < 0.01. ns, no significant

Given miR-548k modulating ESCC lymphatic metastasis through ADAMTS1 and KLF10-EGFR signaling, we asked the question that whether the combination of miR-548k and its effector genes can better predict survival than either molecule. To probe this question, we examined the expression level of these genes in the same cohort by immunohistochemistry assay (IHC). The expression level of KLF10 was inversely correlative to miR-548k and EGFR, while miR-548k was significantly positive correlation with EGFR. The expression level of ADAMTS1 was inversely correlative to miR-548k as well (Fig. 7a, b, Additional file 2: Figure S11a). We then sought to evaluate the clinical relevance of different combination of these associated molecules and found that the classifiers including KLF10 expression level, miR-548k(+)/KLF10(−) (miR-548k high expression and KLF10 low expression), EGFR expression level, miR-548k (+)/EGFR (+) (miR-548k and EGFR both high expression), KLF10 (−)/EGFR (+) (KLF10 low expression and EGFR high expression), miR-548k (+)/KLF10 (−)/EGFR (+) (miR-548k high expression and KLF10 low expression and EGFR high expression), ADAMTS1 expression level, miR-548k (+)/ADAMTS1 (−) (miR-548k high expression and ADAMTS1 low expression) were all significantly correlated to patient overall survival (Fig. 7c, Kaplan–Meier survival analysis and log-rank test). Most of these classifiers were significantly association with lymph node metastasis (Additional file 1: Tables S8-S15 and Additional file 2: Figure S11b). Multivariate Cox regression survival analysis adjusting for age, vascular invasion, T stage, LNM, pathological grade, gender and these classifiers consistently reported strong correlation between these classifiers and shorter survival (Additional file 1: Table S16 and Additional file 2: Figure S11c), indicating that these classifiers except for ADAMTS1 and miR-548k (+)/ADAMTS1 (−) classifiers were potentially servered as independent prognostic factors for outcome in ESCC. In fact, the classifiers miR-548k(+)/KLF10(−), miR-548k (+)/EGFR (+), KLF10 (−)/EGFR (+) and miR-548k (+)/KLF10 (−)/EGFR (+) can be better prognostic factors than either protein as the HR were larger than others (Additional file 1: Table S16 and Additional file 2: Figure S11c). These miR-548k-relative classifiers also showed similar or significantly higher prognostic accuracy than any clinic-pathological risk factor or single miR-548k (Additional file 2: Figure S11d). Taken together, these data suggest that combined miR-548k and its target genes can be used as prognostic biomarkers together.

### The promising application of miR-548k and VEGFC in early diagnosis in ESCC

There were emerging evidences suggested that circulating free miRNAs are an important tool for early stage cancer detection [41]. Thus, we further evaluated the diagnostic value of miR-548k in serum samples of 70 patients with early stage ESCC (T1 stage) and 30 health persons. Interestingly, real time PCR assay revealed that miR-548k abundance was significantly higher than that in serum samples of health persons (Fig. 7d, Student’s t-test, *p* < 0.001). ROC (receiver operator characteristic) curve was used to determine the diagnostic accuracy of miR-548k in this cohort and the result indicated that miR-548k has a potential diagnostic value in ESCC early diagnosis where the AUC was 0.760 with 95% CI (0.667~ 0.853). Additionally, we also found that miR-548k abundance were higher in patients with lymph node metastasis and patient with poor differentiation ESCC (Fig. 7f, g).

To further validation, we enrolled a validation serum cohort from 20 health donors and 56 ESCC patients (including 20 T1 stage, 20 T2 stage and 16 T3 stage patients). The results confirmed that the miR-548k abundance was significantly higher in patients with ESCC. We also observed that the serum level of miR-548k was higher in advanced patients and patient with lymph node metastasis (Additional file 1: Table S18 and Additional file 2: Figure S12a-c). Of note, here we figured out that miR-548k was associated with VEGFC regulation and previous studies also demonstrated that serum VEGFC could serve as a possible diagnostic marker for some cancers [42, 43]. Interestingly, our data also revealed that the serum protein level of VEGFC was significantly higher in advanced patients and patient with lymph node metastasis (Additional file 1: Table S18 and Additional file 2: Figure S12d-f). Pearson’s correlation analysis indicated a weak positive correlation between serum level of miR-548k and VEGFC (Pearson *r* = 0.2108, *p* = 0.0676, Additional file 2: Figure S12 g). Importantly, the diagnostic potentiality of miR-548k was similar to the previous cohort in early ESCC diagnosis (AUC = 0.717, *p* = 0.019), while was shown better diagnostic accuracy in the cohort with all different pathological stages of ESCC patients (Additional file 2: Figure S12 h, i). Similarly, the diagnostic value of serum VEGFC level was almost the same as miR-548k (Additional file 2: Figure S12 h, i). Collectively, the data raised the possibility that detection of miR-548k or VEGFC by liquid biopsy might be a promising tool for ESCC early diagnosis.

## Discussion

As cancer cohorts become larger, analyses such as this will become more powered, raising the opportunity to reevaluate the cancer genome landscape and discover more rational alterations, which will guide clinical practice. In this study, we determined the landscape of SCNAs of ESCC by joint analysis of our previous sequencing data and other Chinese ESCC cohorts. To our knowledge, this was the largest sample size in SCNAs profiling of ESCC. Importantly, we identified 76 significant SCNAs and reinforced the pivotal role of 11q13.3 and 8p23.1 alterations in ESCC, which were the most significantly amplified and deleted regions respectively. Meanwhile, by inferring to the TCGA ESCC cohort of SCNAs in accordance with mRNA expression genes, we figured out a panel of 257 genes that harbored in SCNAs in our pooled cohort. Given that SCNAs might function through changing the expression level of their encompassed genes, these panel of genes should play critical roles in ESCC tumorigenesis. Additionally, there were 28 genes were estimated to inference the lymph node metastasis of ESCC, which including well elucidated genes such as *FADD, PPFIA1, CTTN, TNFSF10* and uncharacterized noncoding genes *FLJ42393, LOC100131635*.

Accumulating evidence has demonstrated the importance of lymphangiogenesis and lymphatic metastasis in turmor progression [4, 35, 36, 44]. Regional lymph node metastasis is well accepted prognostic and diagnostic factor in patients with ESCC, which was considered as an early step for cancer dissemination and progression [35, 45]. However, the mechanisms that control lymph node metastasis are unclear. Amplification of the 11q13.3 region is one of the most common aberrations in multiple human cancers including ESCC and has been implicated with tumor malignancy. Many studies have demonstrated that 11q13.3 amplicon was positively correlated with LNM [1, 19–21, 46], however, despite the clinical relevance, the causal relationship and underlying molecular mechanisms of 11q13.3 amplicon involved in lymphatic metastasis are still unknown. In this study, by setting more rigorous criterions, we found that miR-548k, a microRNA located in the 11q13.3 amplicon, might execute the function of 11q13.3 amplicon to regulate lymphatic metastasis. The miR-548k was most frequently amplified in our cohort and remarkably upregulated in ESCC and its expression level was significantly correlative with patient survival, LNM and AJCC stage. Furthermore, our results also figured out a potential molecular mechanism by which miR-548k may promote cell proliferation, lymphangiogenesis, migration, invasion and lymphatic metastasis in ESCC in vitro and in vivo, via targeting ADAMTS1 and KLF10. Therefore, our results uncover a novel molecular mechanism of 11q13.3 amplicon for tumorigenesis and lymphatic metastasis of ESCC and highlight miR-548k to be the main driving oncogene of this common amplicon.

VEGFR3 is perhaps the most central mediator of lymphangiogenesis [47], and it is regulated by many proteins, including ADAMTS1 [40, 48]. In this study, we proved that miR-548k targeted the 3’-UTR of ADAMTS1 and downregulated its expression. ADAMTS1 was previously found to form a complex with VEGFC and attenuated the phosphorylation of VEGFR3, thus inhibited lymphangiogenesis [40]. Our data suggested that miR-548k overexpression led to the release of more amounts of VEGFC from ADAMTS1 kidnap. Consequently, more secretory VEGFC stimulated phosphorylation of VEGFR3 in lymphatic endothelial cells and promote lymphangiogensis. These tumor microenvironment remodeling effects might be the main contributions for 11q13.3 amplicon to induce lymph node metastasis.

Interestingly, the effect of miR-548k on KLF10 may help to elucidate EGFR overexpression in ESCC. EGFR is one of the most commonly oncogene overexpressed in many cancers, including ESCC [1]. EGFR plays critical roles in modulating signal transduction pathways involved in cell proliferation, cell migration and invasion, angiogenesis [49]. There are about 36.6% to 97% *EGFR* overexpression of ESCC patients [50–57], which is proved to correlate with lymph node metastasis, overall survival and pathologic tumor stages [52, 58, 59]. Intriguingly, compared with the facts that there are more than 50% of ESCC showing EGFR overexpression in protein level, only 15–28% of ESCC specimens exhibited gene amplification [52, 60], which indicates that certain post transcription regulations exist and play a critical role in EGFR associated ESCC malignancies. In the current study, we verified this hypothesis that miR-548k downregulated the EGFR transcriptional suppressor KLF10 and upregulated EGFR level as a consequence.

Importantly, our study identified miR-548k as a promising biomarker for prognosis of ESCC. MiR-548k was significantly upregulated in ESCC, and the expression of miR-548k could be conveniently detected by ISH. Importantly, the strong association between miR-548k upregulation and poor outcome of patients with ESCC has been confirmed by our data. In addition, the prognostic significance of miR-548k CNA was also supported by our previous study [1] and an independent study in ESCC [27]. These interesting results suggested that miR-548k overexpression or gained CNA could be used as prognostic markers in ESCC. Notably, our findings also revealed that the combination of miR-548k and its target genes KLF10 and ADAMTS1 and associated genes EGFR has better prognostic value than either protein, suggesting that miR-548k and its associated genes could be used as prognostic biomarkers together.

It has been well elucidated that circulating free miRNAs are an important tool for early stage cancer detection [41]. In our present study, we have evaluated the abundance of miR-548k in patients with early stage ESCC, advanced ESCC and health persons and found that miR-548k was significantly higher in ESCC patients and shown moderate diagnostic accuracy. More samples from different pathological stages should be subjected to verified the abundance of miR-548k in serum and its diagnostic and prognostic values. Additionally, we noticed that although the abundance of miR-548k was higher in T1 ESCC serum than health people’s, the relative abundance (normalized to U6) was still low, thus detection in exosome might increase the sensitivity and specificity of diagnostic accuracy.

## Conclusions

**Fig. 8:**
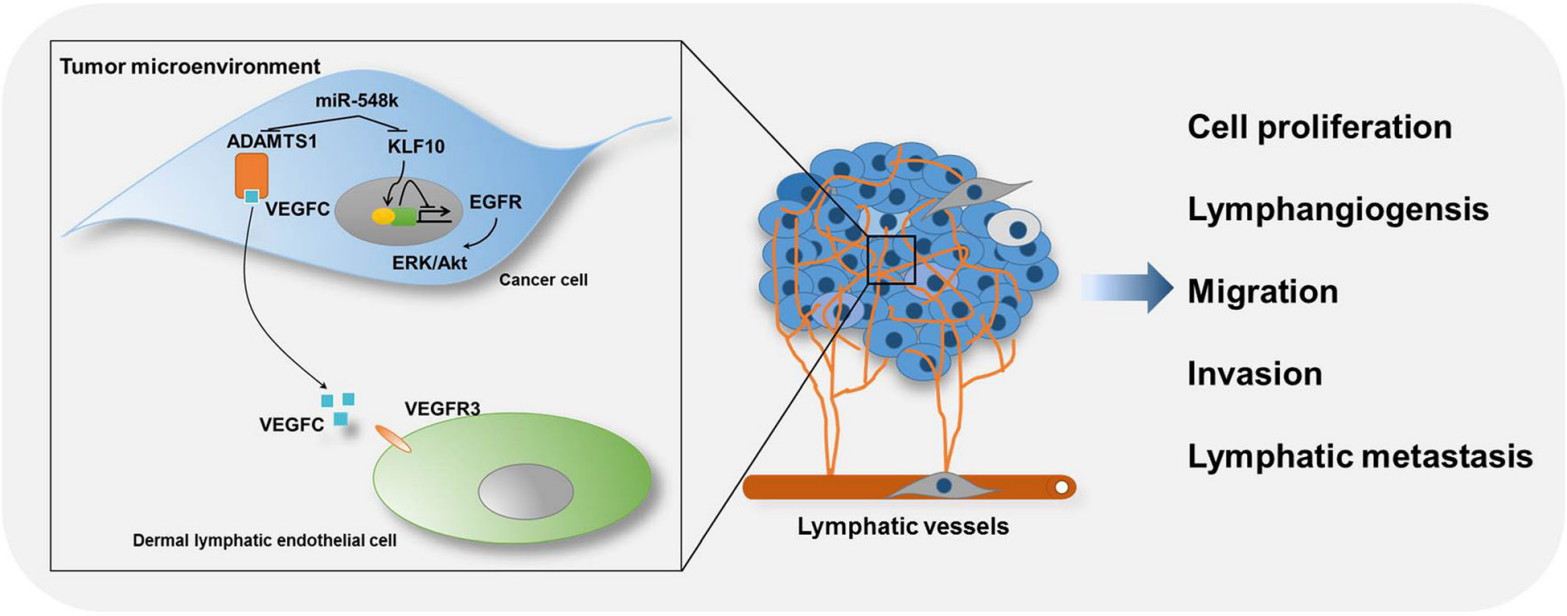
Schematic representation of the miR-548k proposed mode of action in modulating ESCC tumor responses. miR-548k functions as an oncogene in ESCC cells by directly dampening KLF10, a transcriptional repressor of EGFR, thus leading to transcriptional activation of EGFR and subsequently activation of its downstream effectors ERK and Akt, which promote cell proliferation, migration and invasion. In addition, miR-548k functions as a mediator of intercellular communication within the tumor microenvironment by targeting ADAMTS1, resulting in more amount of VEGFC releasing from ADAMTS1 sequestration. Secretory VEGFC stimulates VEGFR3 activation in infiltrated dermal lymphatic endothelial cells and leads to lymphangiogensis and promotes lymphatic metastasis

In summary, we provided extensive characterization of SCNAs in ESCC through a larger cohort, which increases the understanding of the genetic pathophysiology of ESCC. We also proposed a new understanding of 11q13.3 amplicon in implication with ESCC, which is partially depended on diverse functions mediated by miR-548k. In particular, we show that miR-548k regulates different hallmarks of cancer, including cell proliferation, migration, invasion, and tumor microenvironment remodeling through combinatorial regulating of multiple oncogenic routes, involving the ADAMTS1-VEGFC-VEGFR3 pathway and KLF10-EGFR axis (Fig. 8). The recognition of miR-548k in regulating lymphangiogenesis and lymphatic remodelling are functionally important in ESCC progression, which led to the idea that miR-548k might be employed as a new diagnostic and prognostic marker and a predictive marker for anti-VEGFC or anti-EGFR therapy strategies in ESCC treatment, or even served as a therapeutic target synergizing with anti-VEGFC or anti-EGFR therapies.

## Additional files

Additional file 1: Supplementary Tables. (XLSX 157 kb)

Additional file 2: Supplementary Methods and Figures. (DOCX 3297 kb)

## Abbreviations

ADAMTS1: A disintegrin and metalloproteinase with thrombospondin motifs 1
AJCC: American Joint Committee on Cancer
CM: Conditioned media
ESCC: Esophageal squamous cell carcinoma
HDLEC: Human dermal lymphatic endothelial Cell
ISH: In situ Hybridization Histochemistry
KLF10: Krueppel-like factor 10
LNM: Lymph node metastasis
miRNA: microRNA
SCNA: Somatic copy number alteration
TCGA: The Cancer Genome Atlas
