# Supplementary material for: The chromosome 11q13.3 amplification associated lymph node metastasis is driven by miR-548k through modulating tumor microenvironment
Source: Mol Cancer. 2018 Aug 21;17:125. doi: 10.1186/s12943-018-0871-4 (PMC6103855; doi:10.1186/s12943-018-0871-4)
Supplement: Supplementary file 2 — Supplementary Methods and Figures. (DOCX 3297 kb) [file 12943_2018_871_MOESM2_ESM.docx]

**Supplementary Materials and Methods**

**Ethics statement, tissue specimens and clinicopathological characteristics**

Tissue microarrays (TMA) of ESCC specimens were obtained from Shanghai Outdo Biotech Co., Ltd. (SOBC), with the approval of the Institutional Review Board. The detail clinicopathological characteristics of all specimens were summarized in Supplementary Table S6. The patients were followed up 2.8 years to 7.8 years. The ESCC tissues and matched adjacent normal tissues and the serum samples of ESCC patients and health persons used for real time PCR assay were histopathologically and clinically diagnosed at Beijing Cancer Hospital and the Cancer Institute and Hospital, Chinese Academic of Medical Sciences & Peking Union Medical College (Supplementary Table S17). Written informed consent was obtained from all patients prior to the study. The use of the clinical specimens for research purposes was approved by the Institutional Research Ethics Committee.

All animal care and procedures were in accordance with national and institutional policies for animal health and well-being. Mouse experimentations were approved by Cancer Institute and Hospital, Chinese Academic of Medical Sciences & Peking Union Medical College Animal Care and Use Committee. All mouse surgery was performed under anesthesia, and all efforts were made to minimize suffering of animals.

**Cell lines and cell culture**

Esophageal squamous cell carcinoma (ESCC) cell lines KYSE30 and KYSE510, which were kindly provided by Dr. Shimada, Y of Kyoto University, were cultured in RPMI 1640 (Gibco) with 10% fetal bovine serum (FBS). Human dermal lymphatic endothelial cells (HDLECs) were purchased from ScienCell Research Laboratories (Carlsbad, California, USA) and cultured according to the manufacturer’s instructions. All of these cells were maintained at 37°C with 5% CO_2_.

**RNA isolation and quantitative real-time PCR**

Total RNA of ESCC cells was extracted with TRIzol reagent (Invitrogen, Carlsbad, California, USA) and 100 μl serum were applied to RNA isolation by acid phenol-chloroform. The cDNA was synthesized with the PrimeScript RT reagent Kit (Promega, Madison, WI, USA). Real-time PCR was carried out using an ABI 7300 real time PCR system (Applied Biosystems, Foster City, California, USA). Bulge-Loop hsa-miR-548k qRT–PCR Primer Set (Ribobio, miRQ0005882-1-2) was used for the measurement of the relative quantity of hsa-miR-548k. The mRNA expression level of ADAMTS1 was normalized to the endogenous expression of *GAPDH*. Primers were provided by Invitrogen as described below:

*GAPDH* forward, 5’-TCTCTGCTCCTCCTGTTC-3’,

*GAPDH* reverse, 5’-GTTGACTCCGACCTTCAC-3’;

*ADAMTS1* forward, 5’-GGATGGCTGATGTTGGAA-3’

*ADAMTS1* reverse, 5’-CATTAAGGCTGGCACACT-3’

*KLF10* forward, 5’-GAATGGCACCAGACTCTC-3’

*KLF10* reverse, 5’-GATGTGACTCCTTATCCTTGA-3’

**3’- UTR Luciferase Reporter Plasmid Construction**

The 3’-UTR Luciferase Reporter Plasmids of ADAMTS1 were designed and constructed by Shanghai Genechem Co., LTD (Genechem, Shanghai, China). Briefly, the 222 bp 3’-UTR (wild type and miR-548k binding site mutation) of human ADAMTS1 complementary DNA were synthesized chemically and inserted into the luciferase reporter plasmid GV126. Correct sequence of the insert was verified by sequencing.

**Western blot**

After 48 h of transfection, cells were harvested and lysed on ice for 40 minutes in RIPA buffer (10 mM Tris pH 7.4, 150 mM NaCl, 1% Triton X, 0.1% Na-Deoxycholate, 0.1% SDS and 5 mM EDTA) containing Complete Protease Inhibitor Cocktail (Roche Applied Science). The concentration of cellular whole protein was quantified by a colorimetric assay. 60 μg of whole proteins were separated by 8%, or 10% SDS-PAGE gel and then transferred to PVDF membrane. After blocking with 2% bovine serum albumin (BSA), the membrane was incubated with primary antibodies overnight at 4°C. The antibodies used were antibodies to EGFR (Cell Signaling, Boston, MA, USA), Akt (Cell Signaling), Phospho-Akt (Thr308) (Cell Signaling), Erk1/2 (Cell Signaling), Phospho-Erk1/2 (Thr202/Tyr204) (Cell Signaling), KLF10 (Abcam, USA), ADAMTS1 (Abcam, USA), VEGFR3 (Abcam, USA), Anti-Phosphotyrosine (EMD Millipore, USA), VEGFC (Cell Signaling), β-actin (Sigma, St Louis, MO, USA), Secondary antibodies such as goat-anti-mouse IgG (1:2000) and goat-anti-rabbit IgG (1:3000) conjugated with horseradish peroxidase (HRP) were used to probe membrane for 1 h. The membrane was rinsed in 1×PBS with 0.1% Tween. After incubation with the Chemiluminescence substrate, photographs were taken by Image Reader LAS-4000 (Fujifilm) and analyzed by the Multi Gauge V3.2 software.

**Co-immunoprecipitation**

Cells were lysed with buffer (20 mM Tris/HCL, pH 7.6, 100 mM NaCl, 20 mM KCl, 1.5 mM MgCl_2_, 0.5% NP-40) containing Complete Protease Inhibitor Cocktail (Roche Applied Science). Cell lysate was incubated with protein A/G-Sepharose beads preloaded with anti-ADAMTS1 or anti-VEGFR3 antibody. Immunoprecipitates were washed by lysis buffer five times each.

**Immunohistochemistry**

Immunohistochemistry (IHC) analysis was performed and diagnosed by two pathologists blindly on the 185 pair paraffin-embedded ESCC tissue sections (Tissue Microarray) and the xenograft tumor tissues. In brief, the sections were deparaffinized with xylenes and rehydrated in graded ethanol. Sections were submerged into EDTA antigenic retrieval buffer (pH8.0) and microwaved for antigenic retrieval. The sections were then treated with 3% hydrogen peroxide in methanol to quench the endogenous peroxidase activity, followed by incubation with 1% goat serum albumin to block nonspecific binding. The tissue sections were incubated with rabbit anti–EGFR (1:200; Cell Signaling), LYVE-1 (1:200, Abcam) and GFP (1:500, Santa Cruz) overnight at 4°C. After washing, the tissue sections were treated with goat anti-mouse/rabbit IgG HRP-polymer (ZSGB-BIO, Beijing, China) for 20 min. 3, 3’-Diaminobenzidine was used as the chromogen.

The scores were determined by combining the intensity of staining and the proportion of positively stained tumor cells as described in the previous paper (1). First, the intensity was graded as follows: 0, negative; 1, weak; 2, moderate; 3, strong. Second, the proportion of positive tumor cells was graded: 0, <5%; 1, 5–25%; 2, 26–50%; 3, 51–75%; 4,>75%. A final score was derived by multiplication of these two primary scores. Final scores of 0–4 were defined as ‘low expression’ (-); scores of 6–12 as ‘high expression’ (+).

**In Situ Hybridization for miRNA and Immunohistochemistry assay**

In situ hybridization (ISH) for miR-548k was also performed by SOBC using the MiRCURY LNA microRNA ISH Optimization Kit (Exiqon, Vedbaek Denmark). Briefly, the paraffin sections were deparaffinized and treated with proteinase-K (15μg/mL) at 37°C for 10 min. After dehydration, slides were incubated with 100 nM miR-548k locked nucleic acid probe (5’-DIG- AGCAAAATCCGCAAGTACTTTT -3’-DIG) at 50°C for 60 min, followed by stringent washes with 5 standard saline citrate, 1 saline sodium citrate, and 0.2 saline sodium citrate buffers at 50°C; DIG blocking reagent (Roche, Mannheim, Germany) in maleic acid buffer containing 2% sheep serum at room temperature for 15 min; and alkaline phosphatase–conjugated antidigoxigenin (diluted 1:500 in blocking reagent; Roche) at room temperature for 60 min. Enzymatic development was performed by incubating the slides with 4-nitro-blue tetrazolium and 5-brom-4-chloro-3’-Indolylphosphate substrate (Roche) at 30°C for 2h to allow formation of dark-blue 4-nitro-blue tetrazolium formazan precipitate, followed by nuclear fast red counterstain (Vector Laboratories, Burlingame, CA) at room temperature for 1 min. Slides were then dismantled in water, dehydrated in alcohol solutions, and mounted with mounting medium (Vector Laboratories). Scrambled probe and U6 small nuclear RNA–specific probe were used as system control. The scores were determined by combining the intensity of staining and the proportion of positively stained tumor cells as described in the previous paper (1). First, the intensity was graded as follows: 0, negative; 1, weak; 2, moderate; 3, strong. Second, the proportion of positive tumor cells was graded: 0, <5%; 1, 5–25%; 2, 26–50%; 3, 51–75%; 4,>75%. A final score was derived by multiplication of these two primary scores. Final scores of 0–4 were defined as ‘low expression’; scores of 6–12 as ‘high expression’.

**ELISA assay for serum VEGFC measurement**

The serum protein level of VEGFC was detected by human Vascular Endothelial cell Growth Factor C (VEGFC) ELISA Kit (Beijing Bossbio Bio-Technology Co.Ltd, Beijing, China) according to the manufacturer's instructions.

**Supplementary figure legends**

**
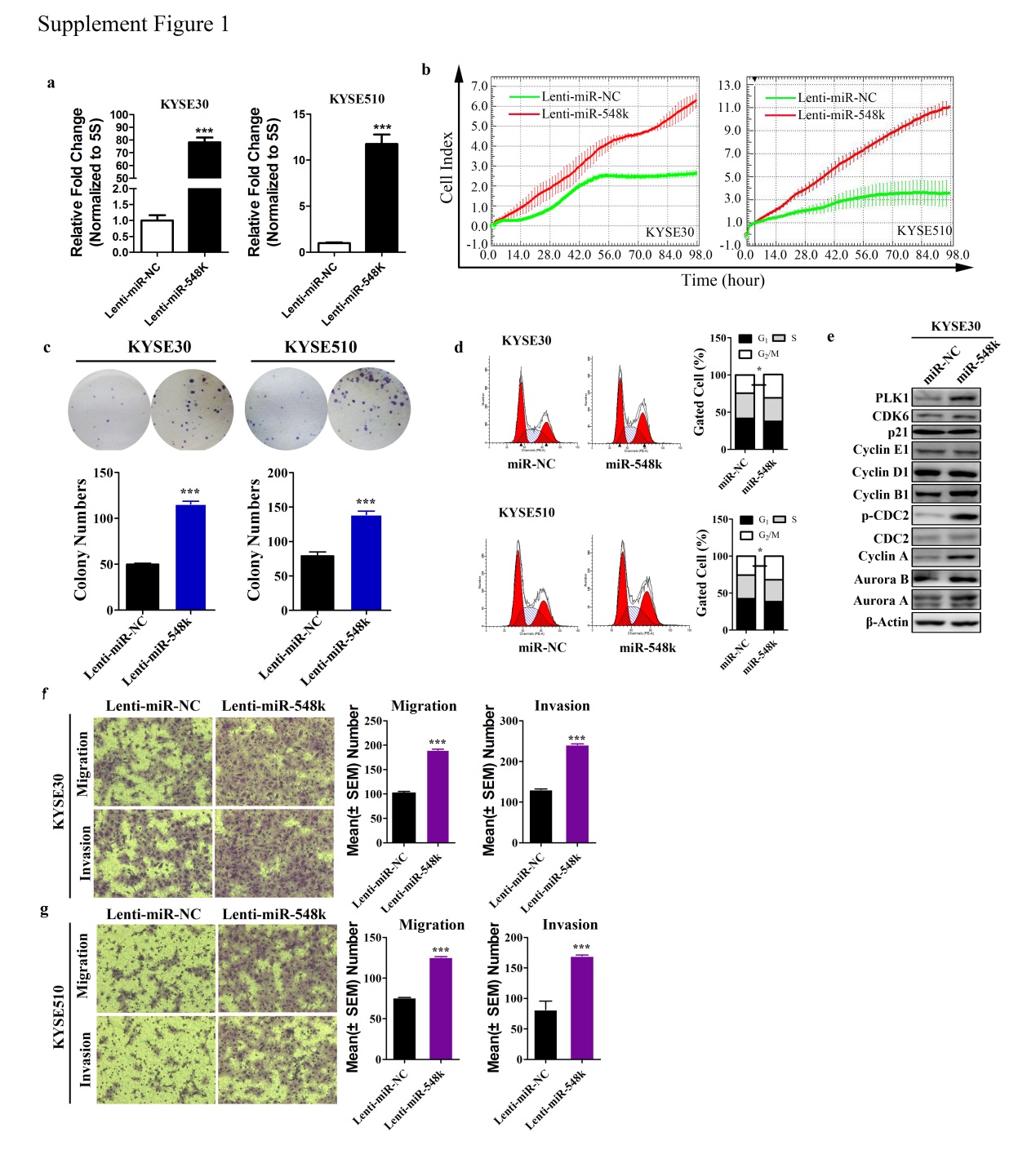
**

**Supplementary Figure 1 Functional studies reveal oncogenic characteristics of miR-548k *in vitro*.** **a**, Stable overexpression of miR-548k by lentivirus in ESCC cell lines (KYSE30 and KYSE510) were measured by real-time PCR. **b**, Growth curves showed that overexpression of miR-548k greatly promoted cell proliferation in KYSE30 and KYSE510 cells. **c**, Overexpression of miR-548k increased colony formation rates of ESCC cells. Representative pictures (Up) and quantitative analysis (bottom) of colony formation assays. **d**, MiR-548k regulated cell cycle distribution in KYSE 30 and KYSE510 cells. **e**, Different cell cycle relative molecules expression levels in miR-548k overexpression cells and control counterparts were examined by Western blot. **f & g**, Overexpression of miR-548k increased cellular motility of KYSE30 and KYSE510 cells. Representative pictures (left) and quantitative data (right) of transwell (migration or invasion) assays. All experiments were performed at least three times and data were statistically analyzed by two-tail t-test. ***p<0.001. Error bars indicate S.E.M.

**
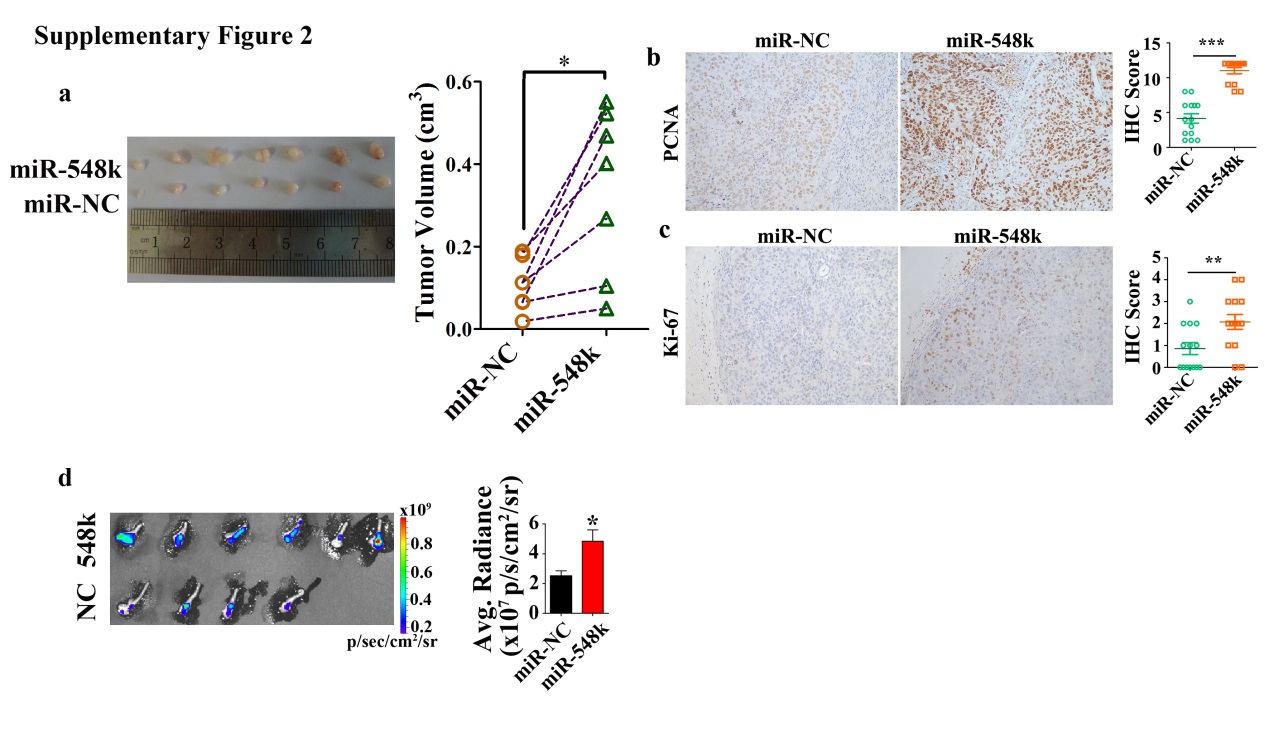
**

**Supplementary Figure 2 Overexpression of miR-548k promotes xenograft tumor formation. a** Stable overexpression of miR-548k in KYSE510 cells enhanced subcutaneous xenograft tumors formation in BALB/c nude mice (n=7). Left, representative picture. Right, quantitative data of the tumor volume after subcutaneous injection for one month. **b & c,** PCNA (a) and Ki-67 (b) expression level in the miR-548k overexpression xenograft tumor group and the control group. Left, representative photos, right, Quantitativ data. **p<0.01. Error bars indicate S.E.M. Scale bar: 500μm or 100μm. **d**, Stable overexpression of miR-548k in KYSE30 cells promoted subserosa tumor growth in esophageal abdominal portion (n= 6 in miR-548k overexpression group and n = 4 in control group). Left, fluorescence imaging of mouse esophageals. Right, quantitative analysis of the GFP radiance.

**
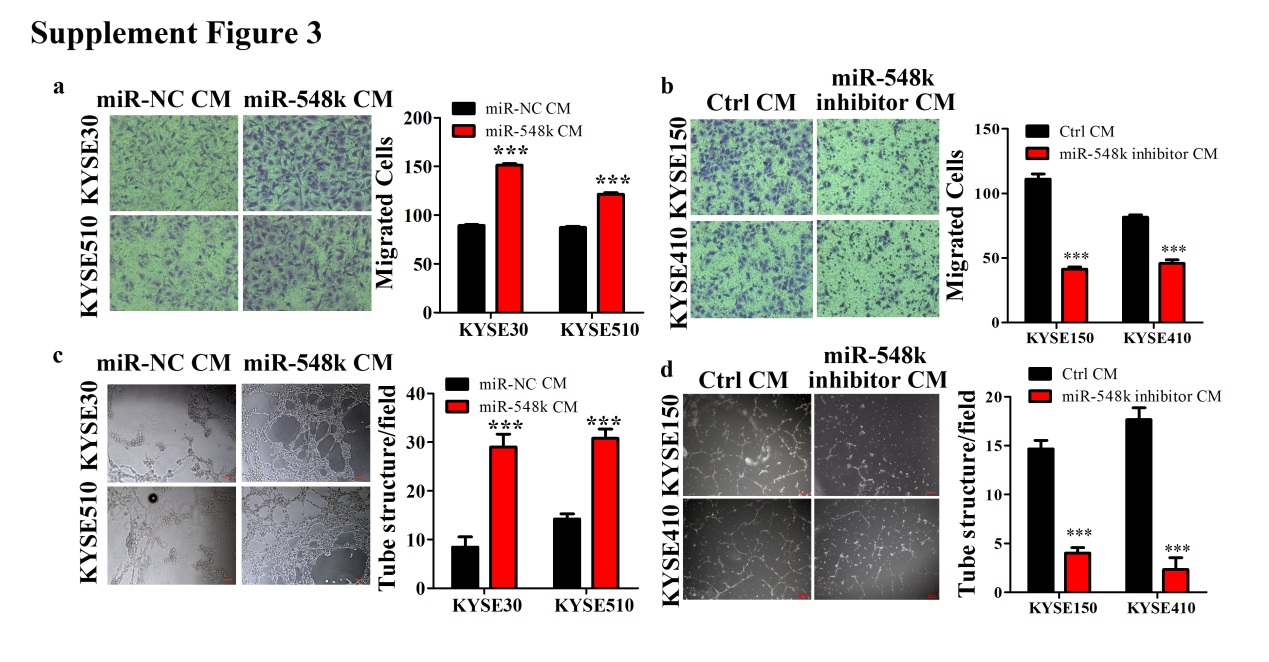
**

**Supplementary Figure 3 MiR-548k promotes esophageal squamous cell carcinoma (ESCC) lymphangiogenesis *in vitro*. a & b**, Transwell migration assays examined the migration ablitiy of of Human Dermal Lymphatic Endothelial Cells (HDLECs) cultured with conditioned medium derived from miR-548k overexpressing (KYSE30-Lenti-miR-548k and KYSE510-Lenti-miR-548k) or silencing (KYSE150 and KYSE410 treated with miR-548k inhibitor) cells and control cells. Left, representative images. Right, quantitative data. **c & d**, Matrigel tube formation assay evaluated the tube formation ablity of HDLECs cultured with conditioned medium derived from miR-548k overexpressing (KYSE30-Lenti-miR-548k and KYSE510-Lenti-miR-548k) or silencing (KYSE150 and KYSE410 treated with miR-548k inhibitor) cells and control cells. CM, conditioned medium. Error bars represent the mean ±S.E.M from three independent experiments, ***p<0.001.

**
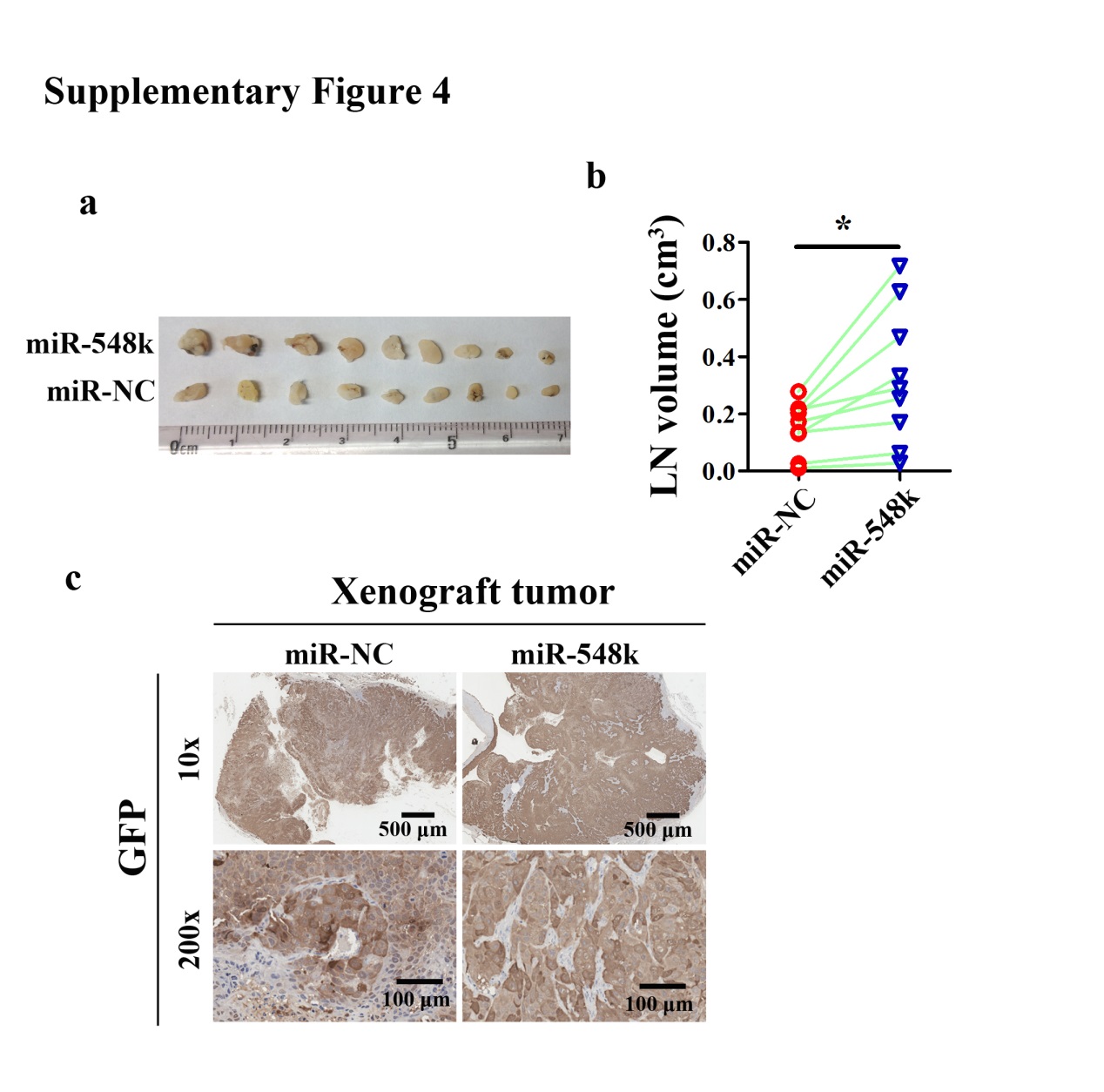
**

**Figure 4 MiR-548k promotes lymph node metastasis in vivo. a & b** Representative images (a) and quantitative analysis of the popliteal lymph node volumes (b), *p<0.05, paired t-test. **c**, GFP positive in the miR-548k overexpression xenograft tumor group and the control group. Scale bar: 500μm or 100μm.

**
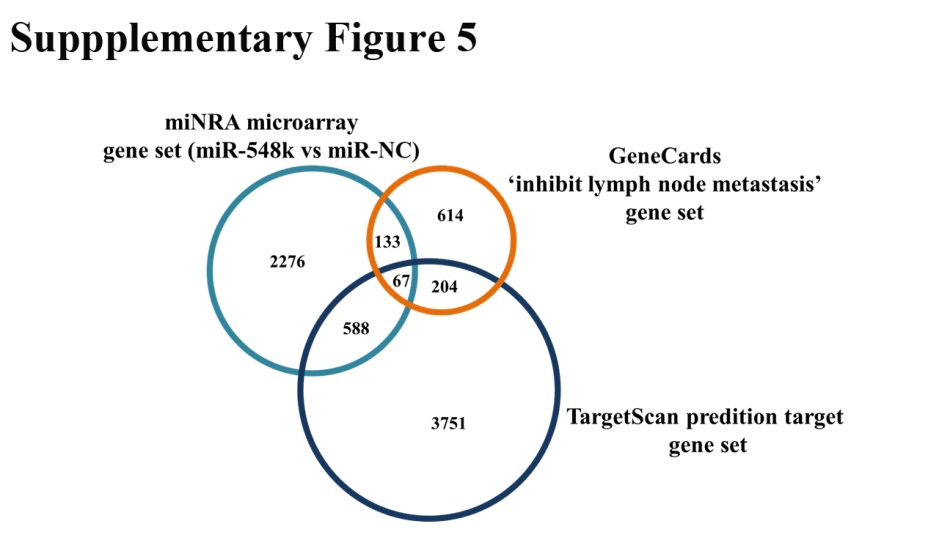
**

**Supplementary Figure 5 Prediction of miR-548k targets.** To screening the potential targets of miR-548k that associated with lymph node metastasis, gene set of target prediction by TargetSca and gene set from mRNA microarray assay on miR-548k overexpression cells and the control cells and gene set obtained from the GeneCards database (http://www.genecards.org) with keyword ‘inhibit hlymph node metastasis’ were overlapping to yield a panel miR-548k target candidates.

**
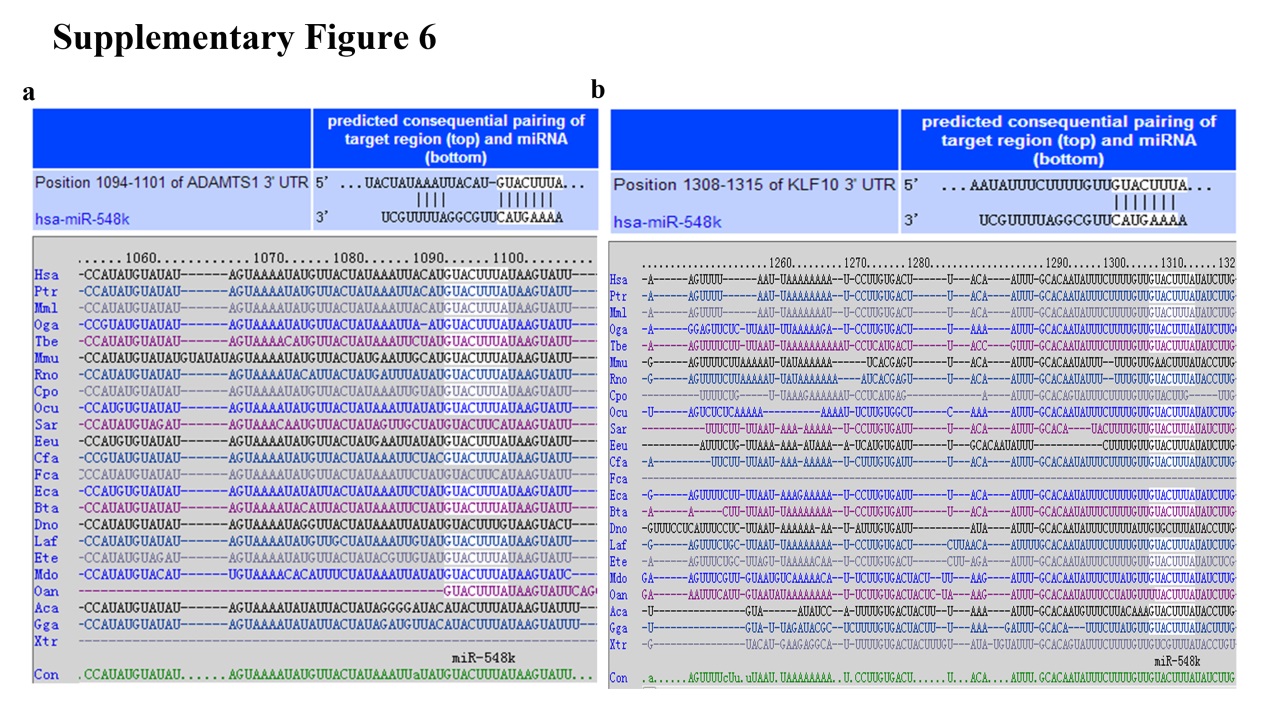
**

**Supplementary Figure 6 TargetScan (**[**http://www.targetscan.org**](http://www.targetscan.org)**) online tool reveals that the seed region of miR-548k in ADAMTS1 (a) and KLF10 (b) genes is conservative across different species.**

**
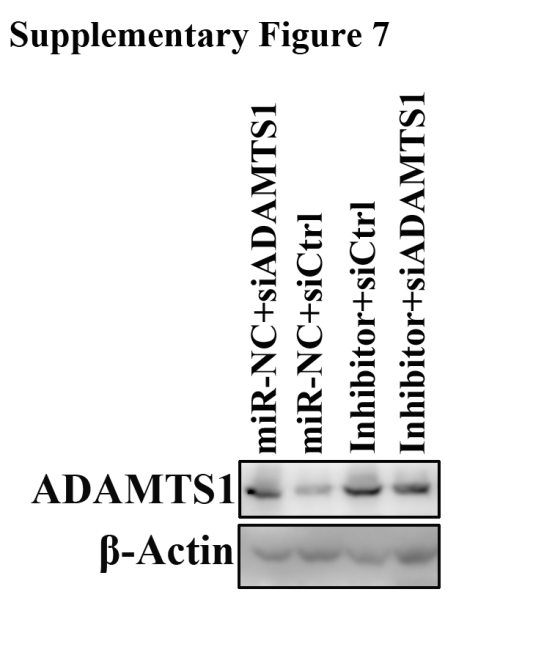
**

**Supplementar Figure 7 Western blot examined the expression level of ADAMTS1 after treating with ADAMTS1 siRNA with or without miR-548k inhibitor.**

**
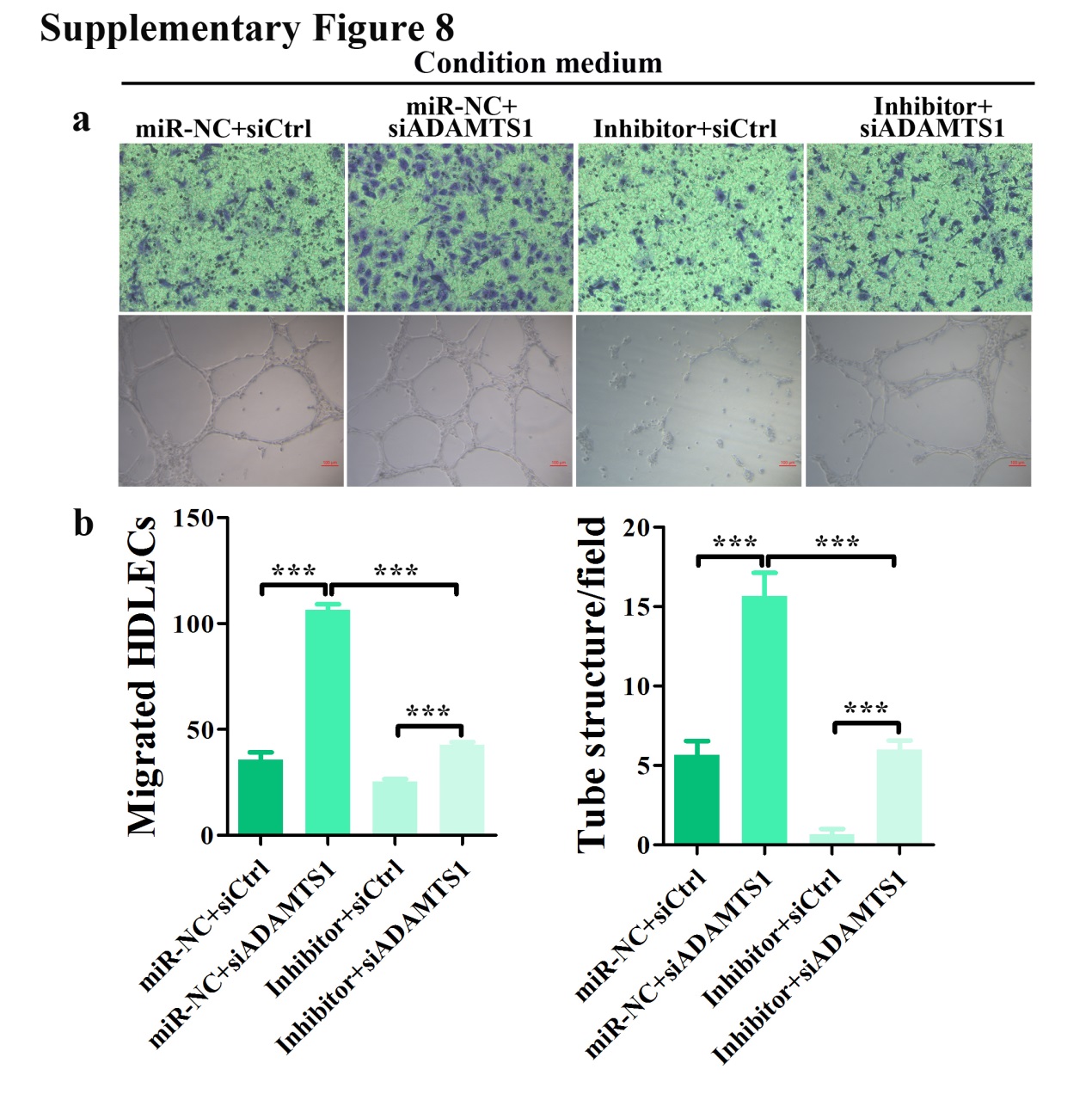
**

**Supplementary Figure 8 Inhibition of miR-548k or knockdown of ADAMTS1 attenuated esophageal squamous cell carcinoma (ESCC) lymphangiogenesis *in vitro*. a & b**, Representative images (a) and quantitative data (b) of Human Dermal Lymphatic Endothelial Cells (HDLECs) cultured with conditioned medium derived from miR-548k inhibited with or without ADAMTS1 silencing in KYSE150 and control cells by transwell migration assays and matrigel tube formation assay. CM, conditioned medium. Error bars represent the mean ±S.E.M from three independent experiments, ***p<0.001.

**
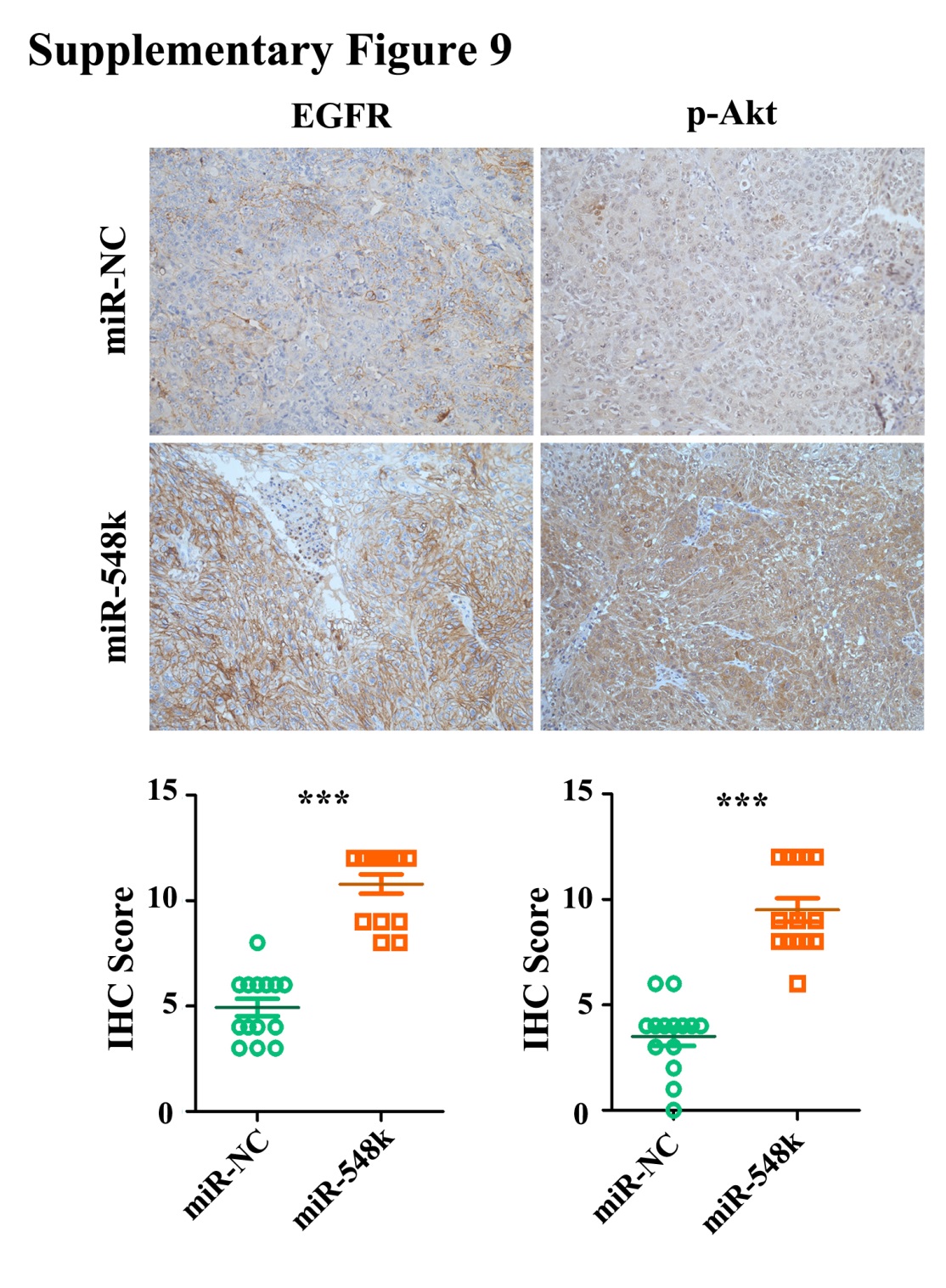
**

**Supplementary Figure 9 EGFR and its downstream effectors Akt were activated in miR-548k overexpression cells formed xenograft tumor tissues.** Up, representative image of EGFR, phospho-Akt (ser 308) IHC images, bottom, quantitative data of the IHC scores of these molecules in miR-548k overexpression cell formed xenograft tumor tissues and their couterparts. Scale bar: 500μm, **p<0.01. Error bars indicate S.E.M.

**
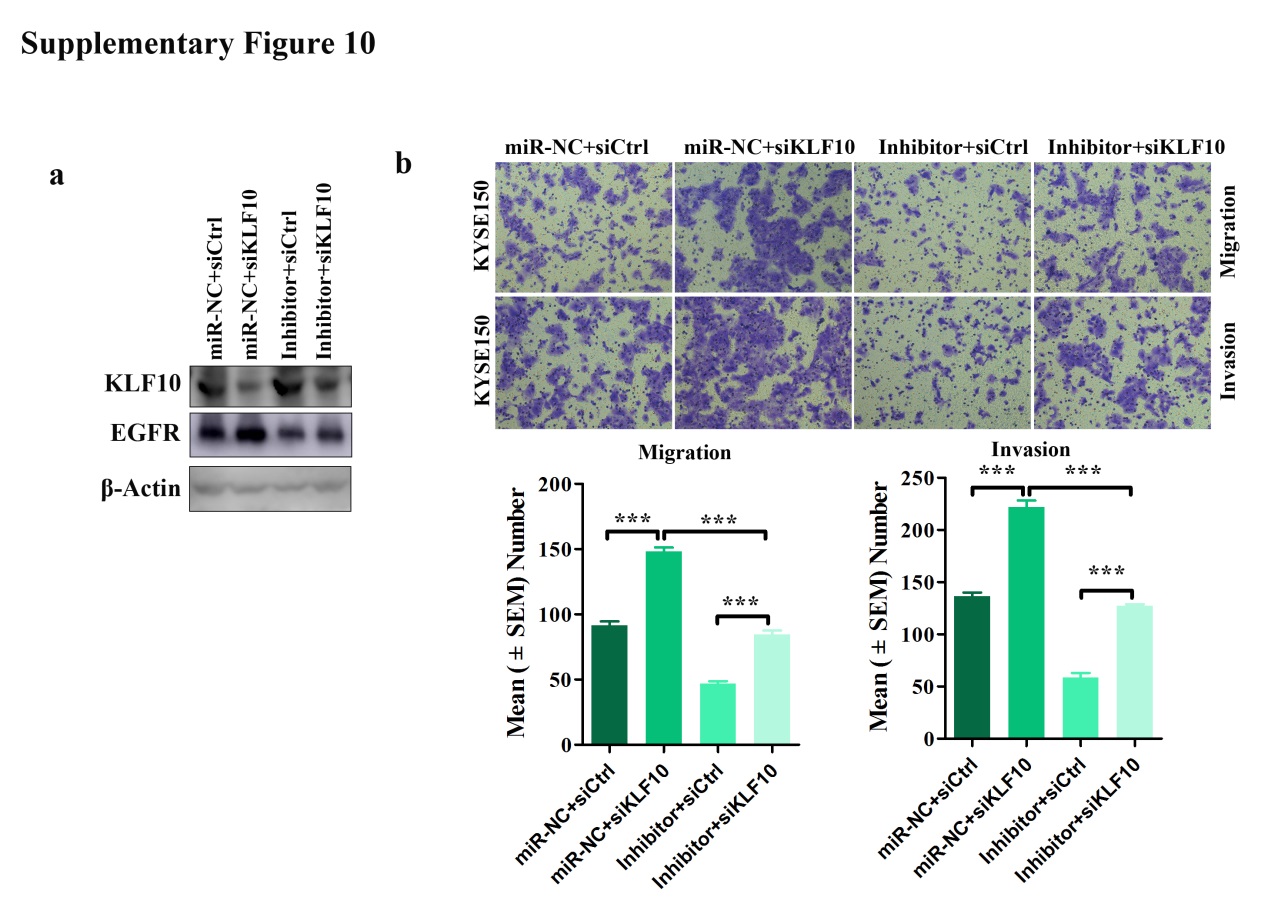
**

**Supplementary Figure 10** **MiR-548k regulated cellular motility through KLF10-EGFR pathway. a**, Western blot analysis the expression of KLF10 and EGFR in KYSE150 cells after siKLF10 or combined with miR-548k inhibitor treatment for 48 hours. **b**, Transwell assays evaluated the migration and invasion capacities under miR-548k inhibition with or without KLF10 silencing. Up, representative images; bottom, quantitative data. Error bars represent the mean ± S.E.M from three independent experiments, ***p<0.001.

**
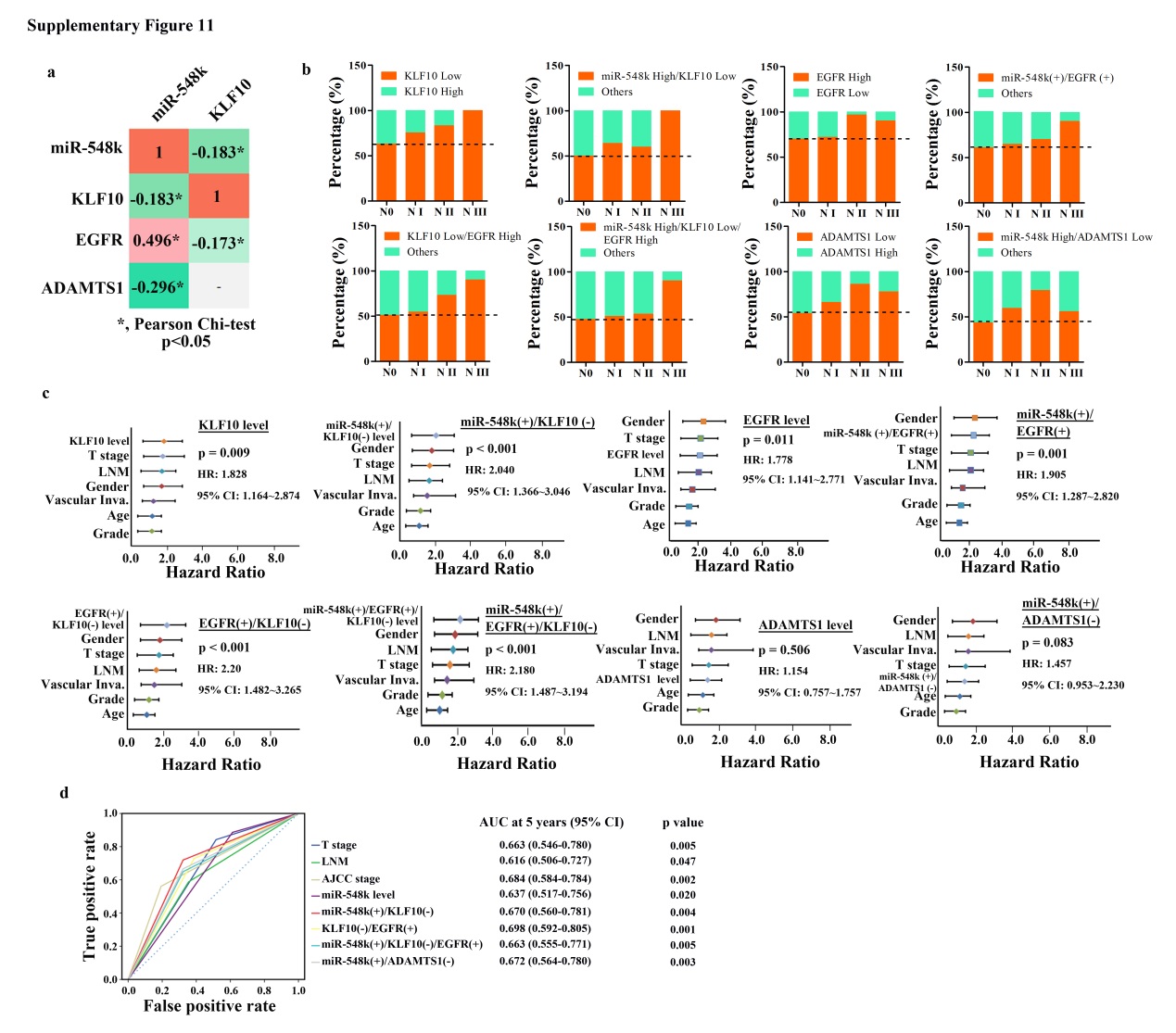
**

**Supplementary Figure 11 The clinical relevance of different classifiers. a**, The correlations between miR-548k and KLF10, EGFR, ADAMTS1, KLF10 and EGFR were analyzed by two-tailed Pearson χ2 test. **b**, The proportion of different miR-548k classifiers in each pathological N stages. The classifier including miR-548k high vs low expression, miR-548k high and KLF10 low expression simutanously vs other patterns, EGFR high vs low expression, miR-548k and EGFR both high expression vs other expression paterns, KLF10 low and EGFR high expression simutanously vs other expression paterns, miR-548k high and KLF10 low and EGFR high expression simutanously vs other paterns, ADAMTS1 low vs high expression, miR-548k high and ADAMTS1 low expression simutanoously vs other expression paterns. **c**, Multivariate Cox analysis of all ESCC patients stratified by different clasifiers as discribed abvoe. 95% CI, 95% confidence interval. HR, hazard ratio. **d**, ROC curves compare the prognostic accuracy of the miR-548k classifier or its associated combination classifiers with clinicopathological risk factors in all 185 patients with ESCC. Comparisons of the prognostic accuracy by the T stages (T1+T2 vs T3+T4), LNM ( lymph node metastasis, yes vs no), AJCC stage (stage 1,2 vs 3), classifiers as indicated. p values show the AUC at 5 years for the miR-548k classifier vs the AUC at 5 years for other features. ROC, receiver operator characteristic. AUC, area under curve.


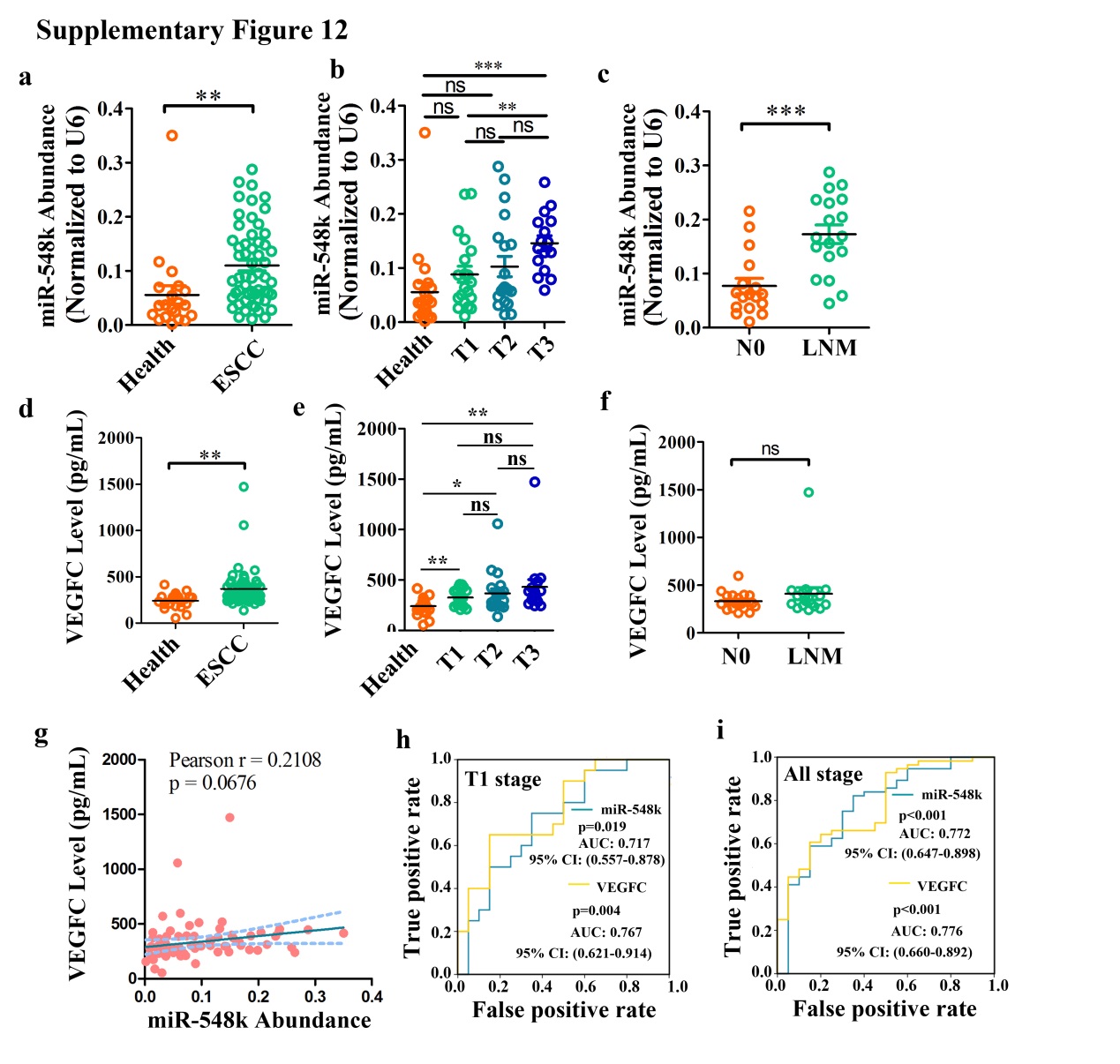


**Supplementary Figure 12 The clinical implication of the serum level of miR-548k and VEGFC in a validation cohort. a**, **b,** The abundance of miR-548k in serum of all ESCC patients (**a**) or different stage ESCC patients (**b**) and health people were detected by real time PCR. Relative expression level were normalized to U6. **c**, MiR-548k abundance in the serum of ESCC patients with or without lymph node metastasis. **d**, **e,** The abundance of VEGFC in serum of all ESCC patients (**d**) or different stage ESCC patients (**e**) and health people were detected by ELISA assay. **f**, VEGFC abundance in the serum of ESCC patients with or without lymph node metastasis. **g**, Pearson's correlation was used to analysis the relationship between miR-548k and VEGFC in the validation cohort. **h** ROC curve evaluated the diagnostic accuracy of miR-548k and VEGFC abundance in the sub-cohort of T1 stage ESCC patients and health persons. **i**, ROC curve evaluated the diagnostic accuracy of miR-548k and VEGFC abundance in the all ESCC patients and health persons. ROC, receiver operator characteristic. AUC, area under the curve. ***p < 0.001. **p < 0.01, *p<0.05. ns, no significant.
